# Associations of Combined Lifestyle Factors with MAFLD and the Specific Subtypes in Middle-Aged and Elderly Adults: The Dongfeng-Tongji Cohort Study

**DOI:** 10.3390/nu15214588

**Published:** 2023-10-28

**Authors:** Hongxia Li, Zhiqiang Cao, Jingxi Li, Lei King, Zhuangyu Zhang, Ying Zhao, Siyi Zhang, Yajing Song, Qian Zhang, Liangkai Chen, Yuhan Tang, Lingling Dai, Ping Yao

**Affiliations:** 1 Department of Nutrition and Food Hygiene, School of Public Health, Tongji Medical College, Huazhong University of Science and Technology, 13 Hangkong Rd, Wuhan 430030, China; lihongxia3697@163.com (H.L.); d202281798@hust.edu.cn (Z.C.); lijingxi_2019@163.com (J.L.); d202281802@hust.edu.cn (L.K.); 15703079104@163.com (Z.Z.); stuzhaoying@163.com (Y.Z.); zsy341kelsey@163.com (S.Z.); m202275594@hust.edu.cn (Y.S.); m202275596@hust.edu.cn (Q.Z.); clk@hust.edu.cn (L.C.); tyh043@126.com (Y.T.); 2 Hubei Key Laboratory of Food Nutrition and Safety, School of Public Health, Tongji Medical College, Huazhong University of Science and Technology, 13 Hangkong Rd, Wuhan 430030, China; 3 Experimental Teaching Center of Preventive Medicine, School of Public Health, Tongji Medical College, Huazhong University of Science and Technology, 13 Hangkong Rd, Wuhan 430030, China

**Keywords:** MAFLD, combined lifestyle, BMI, lifestyle improvement

## Abstract

Metabolic dysfunction-associated fatty liver disease (MAFLD) is the crucial pathogenesis for intra-hepatic and extra-hepatic diseases, especially in elderly adults. Lifestyle management may be a modifiable cost-effective measure for MAFLD prevention, but the evidence is limited. A total of 23,408 middle-aged and elderly individuals were included in a longitudinal study from 2008 to 2018. Combined lifestyle scores (range 0–6) were evaluated by BMI, smoking, drinking, diet, physical activity, and sleep. Logistic regression models were used to calculate ORs for the risks of MAFLD and specific subtypes. The mean age of participants was 61.7 years, and 44.5% were men. Compared with poor lifestyle (scores 0–2), ORs (95% CIs) of the ideal lifestyle (scores 5–6) were 0.62 (0.57–0.68) for MAFLD, 0.31 (0.28–0.34) for MAFLD with excess weight and obesity, 0.97 (0.75–1.26) for MAFLD with diabetes, and 0.56 (0.51–0.62) for MAFLD with metabolic dysregulation. Additionally, lifestyle improvement was associated with lower risks of MAFLD (OR, 0.76; 95% CI, 0.68–0.86), MAFLD with excess weight and obesity (OR, 0.72; 95% CI, 0.63–0.81), MAFLD with diabetes (OR, 0.74; 95% CI, 0.54–1.02) and MAFLD with metabolic dysregulation (OR, 0.49; 95% CI, 0.43–0.55), respectively. Our findings suggest that adherence to a combined healthy lifestyle was associated with lower risks of MAFLD, particularly in excess weight/obese individuals or those with metabolic dysregulation.

## 1. Introduction

Nonalcoholic fatty liver disease (NAFLD) has increased in prevalence, such that nearly 1 billion people globally are affected, which substantially impacts health issues and places a heavy economic burden on individuals, families, and healthcare systems [1]. In 2020, an international panel of 30 experts from 22 countries proposed a new term for NAFLD, metabolic dysfunction-related fatty liver disease (MAFLD), with broader diagnostic criteria [2]. The new criteria focus on the role of metabolic abnormalities in the presence of fatty liver in an inclusive way, so excessive drinking and other related fatty liver are no longer emphasized [3].

MAFLD led to a hot debate when it was proposed. In a nationwide cohort study, 9,584,399 participants aged 40–46 years were included between 2009 and 2010 in Korea, and it showed that the prevalence of MAFLD was up to 37.3%, much higher than that of NAFLD (28.0%) [4]. Additionally, Wong and colleagues reported that the overall prevalence of MAFLD was 34.8% in America, and the prevalence was highest among individuals aged >60 years, rising from 23.2% among individuals aged 18–39 years to 43.8% [5], suggesting more efforts should be made for MAFLD in elderly adults. Intriguingly, a study from Japan suggested that the newly proposed definition of MAFLD better identifies individuals with fatty liver and significant fibrosis than does the term NAFLD, and this definition also better identifies people at high risk of CVD and other adverse cardiometabolic outcomes [4,6,7].

Thus, given the key role of MAFLD in intra-hepatic and extra-hepatic chronic diseases, more attention should be devoted to identifying cost-effective measures for MAFLD prevention in elderly adults. Lifestyle management has been recommended as a fundamental strategy for MAFLD treatment [8,9]. It was reported that routine late-night meals and higher daily alcohol intake were identified as independent lifestyle predictors of MAFLD development [10]. Taheri and colleagues found that an anti-inflammatory diet was inversely associated with MAFLD [11]. Obesity was assumed to be a strong predictor for MAFLD, and weight loss decreased insulin resistance and subsequently increased insulin-like growth factor 1 and improved MAFLD in Caucasian children [12]. Several studies have noted the importance of single lifestyle factors like aerobic exercise, sleep factors, and air pollution in the risk of MAFLD [9,13]; however, evidence about the effects of combined lifestyle factors on MAFLD and specific subtype prevention among elderly individuals is still limited. Owing to the different genetic susceptibilities and lifestyles among diverse ethnic populations, whether such associations persist or are modified by combined lifestyle factors in elderly Asian populations remains unknown.

Therefore, this study aimed to investigate the associations of combined lifestyle factors with MAFLD and specific subtypes among elderly individuals in a large prospective Asian population cohort.

## 2. Materials and Methods

### 2.1. Study Population

The Dongfeng-Tongji (DFTJ) cohort study is an ongoing prospective cohort, and detailed information has been described elsewhere [14]. DFTJ was launched in 2008, and 27,009 retired employees from Dongfeng Motor Corporation were recruited and completed baseline questionnaires and medical examinations between September 2008 and June 2010. Among the participants, 25,978 individuals (96.2%) completed the follow-up in 2013. During the first follow-up in 2013, 14,120 retired workers were newly recruited, consequently, there was a total number of 41,129 participants with baseline information. We completed the second follow-up survey in 2018, and the detailed information is presented in the additional file: Supplementary Figure S1.

In this study, all research was conducted following both the Declarations of Helsinki and Istanbul; all research was approved by the appropriate ethics and institutional review committees; all participants provided written informed consent, and our study was approved by the Ethics and Human Subject Committee of Tongji Medical College, Huazhong University of Science and Technology.

### 2.2. Definition of Lifestyle Factors and Scores

The definition and scoring of healthy and unhealthy lifestyle factors are summarized in the additional file: Supplementary Table S1. Briefly, smoking status was defined as noncurrent smokers (never smoking), former smokers, and current smokers, and the low-risk group was defined as noncurrent smokers. We defined participants who reported never drinking alcohol as being at low risk for alcohol consumption status in our present study.

Total duration per week was calculated as duration (hours per time) × frequency (times per week). A median of 150 min per week was set as the cutoff [15]. Participants who engaged in a higher physical activity level were defined as being in the low-risk group. As a previous study reported, the dietary factor was emphasized in three food items, including vegetables, fruits, and meat, which were addressed in the 2019 American College of Cardiology/American Heart Association guideline on lifestyle management to reduce cardiovascular risk [15,16]. Given that cardiovascular disease and MAFLD share some common risk factors, accordingly, we defined those participants who ate vegetables and fresh fruits every day but meat less than daily as the low-risk group. Generally, adiposity was measured by BMI, and we defined the BMI of 18.5 to 23.9 kg/m^2^ as the low-risk group, which was based on the standard classification specific to Asians [17]. Considering the J-shaped association between sleep duration and the risk of MAFLD, nighttime sleep duration was grouped into optimal (7–9 h/day) and not optimal (<7 or >9 h/day).

A favorable lifestyle was assessed based on the above six lifestyle factors, coded 1 point to participants for the low-risk factors. Overall, lifestyle was subsequently categorized into poor (lifestyle score ≤ 2), intermediate (lifestyle score is 3 or 4), and ideal (lifestyle score ≥ 5) groups.

### 2.3. Ascertainment of Baseline and Incident MAFLD

According to the latest consensus proposed by a panel of international experts from 22 countries and the diagnostic criteria recommended by the Asian Pacific Association for the Study of the Liver [2,18], MAFLD was defined as the presence of hepatic steatosis (diagnosed based on B ultrasound) with one or more of the following: (1) BMI ≥ 23 kg/m^2^ in Asians; (2) T2DM; or (3) at least two MD described in the additional file: Supplementary Table S2. In this study, specific MAFLD subtypes were defined as MAFLD with excess weight or obesity (BMI ≥ 24 kg/m^2^), diabetes, or ≥2 MD.

### 2.4. Statistical Analysis

We used frequencies (percentages) to describe categorical variables and means ± standard deviations (SD) to describe continuous variables. Continuous variables used the two-sample independent *t*-test, and categorical variables used the Chi-squared test to compare the differences between groups.

The univariate and multivariable-adjusted odds ratios (ORs) with a 95% confidence interval (CI) of lifestyle for MAFLD and its subtypes were examined using logistic regression analysis. As long as the 95% CIs for the OR do not contain 1, the *p* value is less than 0.05, which means that the relationship between *p* value and 0.05 can be judged according to whether the 95% confidence interval contains 1 or not. Therefore, the *p* value is not given again in our study. Lifestyle scores were categorized into poor (0–2), intermediate (3–4), and ideal (5–6) groups; the reference group was the poor lifestyle category. We used three models to assess the associations of lifestyle scores with MAFLD and the specific subtypes. In model 1^a^, we adjusted for age (continuous), sex (male or female), and educational attainment (less than high school, high school or equivalent, or college or above). In model 2^b^, we further adjusted for hypertension status (yes or no), hyperlipidemia (yes or no), CVD (yes or no), and diabetes (yes or no). In model 3^c^, we additionally adjusted for LDL-C (continuous) and TC (continuous).

Furthermore, to assess the associations of lifestyle changes with the risks of MAFLD and its subtypes, we analyzed 10,960 participants who completed baseline and subsequent follow-up surveys in 2013. Lifestyle changes were defined as the changes from the baseline to the first follow-up, due to samples being divided into nine groups (poor to the poor group, intermediate to the poor group, ideal to the poor group, poor to intermediate group, intermediate to intermediate group, ideal to intermediate group, poor to the ideal group, intermediate to the ideal group, ideal to ideal group, respectively) which were very uneven, we combined the poor group and intermediate group as the low-scoring group for subsequent analyses, which categorized participants into four groups: consistently low, high to low, low to high, consistently high, and set the consistently low as the reference.

Moreover, several secondary and sensitivity analyses were conducted; detailed information is presented in the Supplementary Materials. A restricted cubic spline model with 3 knots (10th, 50th, 90th) was utilized to test the dose–response association between lifestyle score and incident MAFLD or specific subtypes among the elderly participants.

The important covariates were the stratified factors. Potential interactions between lifestyle and stratification factors were evaluated by introducing a multiplicative term between lifestyle and stratification variables as continuous variables into the multivariate models, and testing whether the coefficient of the interaction term was equal to zero. Taking increased false positives in multiple hypothesis testing into account, we adjusted the *p* value with the Bonferroni correction. We also calculated the false discovery rate (FDR) to identify as many significant interactions as possible while controlling for a relatively low proportion of false positives; FDR < 0.05 was considered significant. Moreover, several secondary analyses were conducted. Firstly, evaluating multiple lifestyle factors with equal weight might limit us to concluding individual risk factors, and lifestyle score calculated according to the actual weight of each lifestyle factor is more appropriate [15]. Therefore, weighted lifestyle scores based on the β coefficients of each lifestyle factor in the logistic regression model were conducted to highlight the more important factors in this study, weighted lifestyle score was included in models as categorical [19]. Secondly, stratified analyses were conducted by age (<65 or ≥65 years), sex (male or female), hypertension (yes or no), and hyperlipidemia (yes or no). Thirdly, we further assessed the association of five different lifestyle factors with outcomes by removing one lifestyle factor each time. Fourthly, we assessed the association of different components of lifestyle factors by including one lifestyle factor each time, based on three lifestyle factors (BMI, smoking, alcohol consumption).

Furthermore, we performed several sensitivity analyses. First of all, after the diagnosis of CVD, participants might change their lifestyle, to reduce possible confounding, we excluded participants with prevalent CVD. Second, we redefined the healthy level of BMI with the criterion of the World Health Organization (WHO). Third, given the potential confounding of psychological factors in association with lifestyle with MAFLD, we further adjusted the mental stress factors in a subset of this study population. Fourth, to exclude the effects of incomplete data, participants with complete data were included for analysis. Finally, we also input missing covariates by multiple imputations to test the association of lifestyle with MAFLD and the specific subtypes.

All statistical analyses were performed with SPSS 25.0 (SPSS Inc, Chicago, IL, USA) and R 4.2.2 (R Project for Statistical Computing) statistical software. The *p* values for all hypotheses tests were two-sided, and *p* < 0.05 was considered statistically significant.

## 3. Results

The baseline characteristics of 23,408 participants (mean (SD) age, 61.7 (7.9) years; 10,408 (44.5%) men) according to lifestyle categories are shown in Table 1. Overall, 4035 (17.2%) participants had a poor lifestyle, 13,236 (56.5%) participants had an intermediate lifestyle, and 6137 (26.2%) participants had an ideal lifestyle. Compared with participants with a poor lifestyle, individuals who adhered to an ideal lifestyle were more likely to be female, younger, leaner, and less likely to develop hypertension, hyperlipidemia, diabetes, and CVD. The characteristics of the participants included and excluded are displayed in the additional file: Supplementary Table S3. The participants’ lab test results at baseline and the end of follow-up are presented in the additional file: Supplementary Table S4.

As shown in Table 2, during a median follow-up of 7.9 years, the risks of developing MAFLD or specific subtypes decreased dramatically with the accumulation of the favorable lifestyle factors (all *p* for trend <0.05 except for MAFLD with diabetes). After adjustment for covariates, compared with individuals with a poor lifestyle, participants with an ideal lifestyle had the lowest risks of MAFLD (OR, 0.62; 95% CI, 0.57–0.68), MAFLD with excess weight or obesity (OR, 0.31; 95% CI, 0.28–0.34), MAFLD with diabetes (OR, 0.97; 95% CI, 0.75–1.26), and MAFLD with MD (OR, 0.56; 95% CI, 0.51–0.62). Moreover, when using a model with the weighted lifestyle score, inverse associations between favorable lifestyle and risks of MAFLD and specific subtypes were broadly similar (additional file: Supplementary Table S5) and the association between the ideal lifestyle and MAFLD with diabetes was strengthened in the weighted model (OR, 0.75; 95% CI, 0.63–0.88).

As shown in the additional file: Supplementary Figure S2, dose–response analyses displayed the inverse linear associations of weighted lifestyle score with risks of MAFLD and MAFLD with diabetes (*p* for non-linear association = 0.099 and 0.155, respectively) and the non-linear associations between weighted lifestyle score and MAFLD with excess weight or obesity and MAFLD with MD. When stratified by age, sex, hypertension status, and hyperlipidemia status, the ideal lifestyle was steadily inversely associated with the risks of MAFLD and specific subtypes (additional file: Supplementary Tables S6–S9).

**Figure 1:**
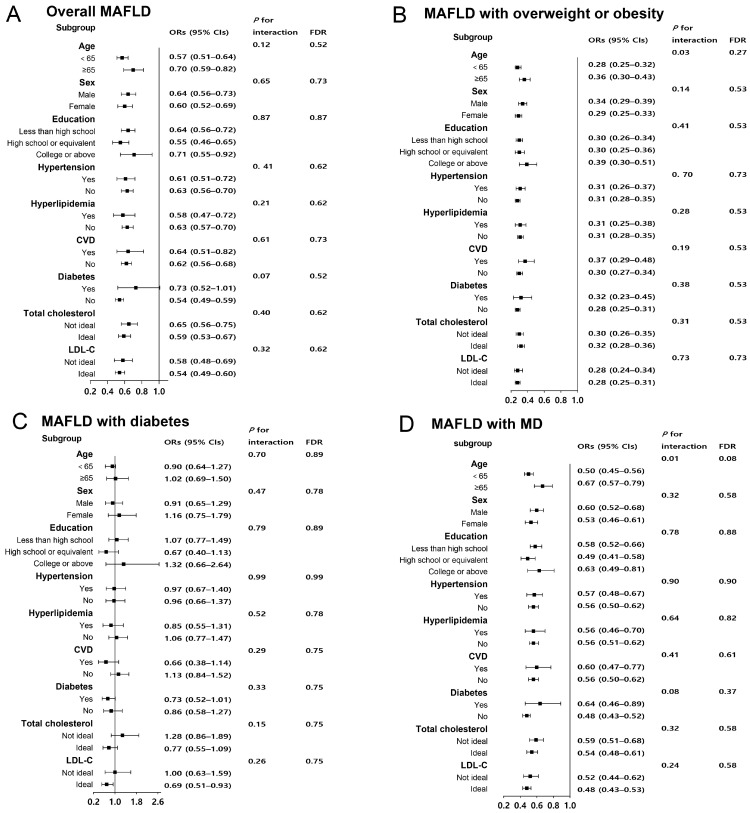
Associations of healthy lifestyle score with the risks of MAFLD and the subtypes in participants stratified by demographic and metabolic features. The dots indicate the ORs of an ideal lifestyle score of 5–6 compared with a poor lifestyle score of 0–2, and the horizontal lines indicate the 95% CIs. Adjusted for age (continuous), sex (male or female) and education (less than high school, high school or equivalent, college or above), hypertension (yes or no), CVD (yes or no), hyperlipidemia (yes or no), diabetes (yes or no), total cholesterol (continuous), and LDL-C (continuous). MAFLD, metabolic dysfunction-associated fatty liver disease; MD, metabolic dysregulation; FDR, false discovery rate; ORs, odds ratios; CIs, confidence intervals.

The potential interactions between lifestyle and stratification factors were evaluated by introducing a multiplicative term between lifestyle and stratification variables. As shown in Figure 1, the associations of lifestyle with MAFLD and specific subtypes seemed to be more evident among participants younger than 65 years old, female, without CVD or diabetes, and with ideal serum LDL-C levels. Still, no interactions between the lifestyle and covariates in MAFLD and specific subtypes were observed. When the lifestyle score was weighted, the associations of lifestyle with MAFLD and specific subtypes were also evident among younger individuals without hyperlipidemia, CVD, or diabetes (additional file: Supplementary Figure S3).

As for the associations of each healthy lifestyle factor with risks of MAFLD and specific subtypes, optimal BMI was related to ORs (95% CI) of 0.44 (0.42–0.47), 0.11 (0.10–0.12), 0.79 (0.67–0.93) and 0.41 (0.38–0.43) for MAFLD, MAFLD with excess weight or obesity, MAFLD with diabetes and MAFLD with MD, respectively (additional file: Supplementary Table S10). In addition, non-drinking was also a negatively correlated with MAFLD and its subtypes (additional file: Supplementary Table S10). A robust association remained for one score increase according to three basic lifestyle factors (additional file: Supplementary Table S11). As shown in the additional file: Supplementary Table S12, when BMI was removed from the lifestyle score, the reserved association was obviously weakened.

As shown in Table 3, compared with individuals with the consistently low lifestyle score during the two follow-up periods, the ORs (95% CI) of participants who changed from the low-scoring to the high-scoring lifestyle were 0.76 (0.68–0.86), 0.72 (0.63–0.81), 0.74 (0.54–1.02) and 0.49 (0.43–0.55) for MAFLD, MAFLD with excess weight or obesity, MAFLD with diabetes and MAFLD with MD, respectively. Moreover, the risk was lowest among participants having a consistently high-scoring lifestyle, and the ORs (95% CIs) were 0.71 (0.61–0.82), 0.64 (0.56–0.74), and 0.41 (0.35–0.48) for MAFLD, MAFLD with excess weight or obesity, and MAFLD with MD, respectively.

The associations remained broadly consistent in sensitivity analyses by excluding participants with prevalent CVD (additional file: Supplementary Table S13), excluding individuals with incomplete information at baseline (additional file: Supplementary Table S14), including mental health for further adjustment (additional file: Supplementary Table S15), redefining the healthy level of BMI with WHO criteria (additional file: Supplementary Table S16), or imputing missing covariates by multiple imputations (additional file: Supplementary Table S17).

## 4. Discussion

In this prospective cohort study of more than 40 thousand middle-aged and elderly Chinese adults, the combined healthy lifestyle was associated with lower risks of MAFLD and specific subtypes. In these healthy lifestyle factors, optimal BMI is one of the key factors in reducing the risks of MAFLD and the specific subtypes. Our findings highlighted that adherence to a favorable lifestyle might benefit lower risks of MAFLD and specific subtypes, particularly in individuals who are overweight/obese or with metabolic dysregulation.

A controlled clinical trial reported that lifestyle interventions of 12-week aerobic exercise could improve BMI, and waist circumference and reduce fibrosis and hepatocyte ballooning in 58% (*p* = 0.034) and 67% (*p* = 0.020) of patients with biopsy-confirmed MAFLD [9]. Combined lifestyle factors (low carbohydrate diet, aerobic training, and resistance training) significantly improved the serum indicators in adults with MAFLD [20]. Still, several limitations restrict the understanding and prevention of MAFLD. First, middle-aged and elderly adults are at high risk of MAFLD, but sufficient attention has not been paid to prevent MAFLD in this specific group. Second, the sample size was too small to address the generalizability of the findings, no information on lifestyle changes, and insufficient adjustment for several important covariates [10,21]. Third, only a single lifestyle (diet, alcohol consumption) was considered, and the effects of combined healthy lifestyle factors on the risk of MAFLD were ignored [11,22]. Last, race is a key factor in MAFLD, but previous studies were based on the American population [5,23], and some evidence of the Asian population should be reported to test the generalizability.

Age and sex are considered risk factors for MAFLD. Data from the Third National Health and Nutrition Examination Surveys (NHANES 1988–1994) showed that MAFLD patients were elderly (48.8 ± 15.1 vs. 46.8 ± 15.8, *p* < 0.001), predominantly male (1959 (50.4%) vs. 2014 (46.3%), *p* < 0.001) compared to NAFLD patients [24]. Furthermore, results from NHANES (2011–2018) showed that MAFLD prevalence increased with age, rising from 23.2% (95% CI, 21.7–24.6%) among individuals aged 18–39 years to 43.8% (95% CI, 42.1–45.5%) when aged >60 years, regardless of their gender, or race/ethnic groups [5]. Among participants aged >60 years, MAFLD prevalence was 47.7% among men and 63.6% among Hispanics [5,22]. Consistent with previous studies, our present study found that the association between a healthy lifestyle and MAFLD was strengthened in elderly (<65 years vs. >65 years: 0.57 (0.51–0.64) vs. 0.70 (0.59–0.82)) and male (male vs. female: 0.64 (0.56–0.73) vs. 0.60 (0.52–0.69)) individuals.

A healthy lifestyle is the cornerstone of steatohepatitis care, and adopting a healthy lifestyle is the most cost-effective strategy for preventing MAFLD-mediated non-communicable diseases [25]. A recent meta-analysis of thirty-four RCT studies with 2652 participants who were all obese (8% with diabetes) found that the healthiest lifestyle was associated with a lower risk of MAFLD [20]. Our present study leveraged data from 23,408 middle-aged and elderly participants with 7.9 years median follow-up duration and found that the healthiest lifestyle was associated with a 32% lower risk of MAFLD compared with the least healthy lifestyle, providing much stronger evidence highlighting management of MAFLD by maintaining a healthy lifestyle. Of note, the associations between combined healthy lifestyle factors and MAFLD with excess weight or obesity and MAFLD with MD were much stronger (0.31 (0.28–0.34), 0.56 (0.51–0.62), respectively), which was independent of age, sex, hypertension, hyperlipidemia, CVD, and diabetes, indicating that favorable lifestyle interventions might be a key management and treatment strategy for hepatic steatosis accompanied by excess weight/obesity or metabolic abnormalities.

Among the modifications of various lifestyles, optimal BMI maintenance has been a widely recognized crucial recommendation for NAFLD treatment [26]. Indeed, a close association between the risk of MAFLD and obesity has been reported: 59.1% of obese patients who underwent a liver biopsy were found to have MAFLD, whereas only 3% to 5% of the general population have the disease [27]. Consistent with this view, our study found that optimal BMI was the most important protective factor for MAFLD and the specific subtypes, including MAFLD with diabetes (OR, 0.79; 95% (0.67–0.93)). In addition, a cross-sectional study examined 11,766 participants and found that among male patients with alcohol consumption of >70 g/week, several noninvasive liver fibrosis scores were significantly higher in the MAFLD group than in the NAFLD group [21]. In the present study, we also found that the key component of a favorable lifestyle—current nondrinking, was inversely associated with the risks of MAFLD and the specific subtypes. Notably, we found that lifestyle improvement was associated with significantly lower risks of MAFLD, MAFLD with excess weight or obesity, and MAFLD with MD, indicating that it is never too late to improve lifestyle for MAFLD prevention.

To our best knowledge, no study has examined the association of combined healthy lifestyle factors with the risk of MAFLD among middle-aged and elderly adult participants. Moreover, we are the first to investigate the association of lifestyle factors combination and lifestyle changes, with the risks of MAFLD and the specific subtypes. The underlying pathogenetic mechanisms for the beneficial associations of a favorable lifestyle with the risk of MAFLD involve various pathways affecting the metabolism of liver fat via regulating visceral fat accumulation, insulin resistance, inflammation, mitochondrial and endoplasmic reticulum dysfunction, and an imbalance in gut microbiota (the so-called “multiple parallel hits” theory) [28]. MAFLD was associated with high risks of incident diabetes (risk ratio, 2.2; 95% CI, 1.7–2.5), CKD (risk ratio, 1.6; 95% CI, 1.4–1.9), and CVD (hazard ratio, 1.4; 95% CI, 1.2–1.8) [29], which might aggravate these extra-hepatic diseases through the above metabolic pathways and molecular mechanisms.

Although the prospective design, large sample size, long-term follow-ups, and standardized variable definition ensure the validity, accuracy, and reliability of our findings, some limitations still need to be noted. First, our findings apply to the middle-aged and elderly Asian population, but these findings require cautious interpretation in other ethnic groups. Second, measurement errors were inevitable in self-reported assessments of lifestyle factors. Third, due to missing information, the characteristics of the participants included and excluded in this study were different, which might cause selection bias. Fourth, the six dimensions of lifestyle might not contain all aspects needed to assess lifestyle, although the major modifiable components were reported in previous literature [19,30]. Fifth, the dietary factor was assessed by a simple food frequency questionnaire without information about food portion size. Therefore, we could not adjust the total energy intake in the models. However, the associations of MAFLD with meat, fruit, and vegetable intake frequency were reported in other sizeable longitudinal studies [31,32].

As shown in the Graphical Abstract, this cohort study of middle-aged and elderly participants found that adhering to a healthy lifestyle was associated with lower risk of MAFLD, MAFLD with excess weight or obesity, MAFLD with diabetes, and MAFLD with metabolic dysregulation among individuals with different metabolic characteristics. Additionally, lifestyle improvement reduced the risks of MAFLD and the specific subtypes. Our findings highlight the urgent need for multi-component lifestyle management among individuals having MAFLD with excess weight or obesity, diabetes, and metabolic dysregulation to avoid intra-hepatic and extra-hepatic diseases, and maintaining optimal body weight should be prioritized.

## Figures and Tables

**Table 1:** Baseline characteristics of participants according to lifestyle score (n = 23,408).

Characteristics	Lifestyle Score			*p* Value
	Poor (0–2)	Intermediate (3–4)	Ideal (5–6)	
No. of cases	4035	13,236	6137	
Age, y	62.0 (7.2)	61.8 (7.9)	61.4 (8.3)	<0.001
BMI, kg/m^2^	24.2 (3.0)	23.5 (2.9)	22.4 (2.2)	<0.001
Waist circumference, cm	83.5 (8.5)	80.8 (8.4)	77.9 (7.5)	<0.001
Male	2748 (68.1)	6005 (45.4)	1655 (27.0)	<0.001
Education attainment, n (%)				<0.001
Less than high school	2356 (58.9)	7679 (58.4)	3380 (55.5)	
High school or equivalent	1110 (27.7)	3890 (29.6)	1948 (32.0)	
College or above	537 (13.4)	1586 (12.1)	757 (12.4)	
Alcohol consumption, n (%)				<0.001
Never	442 (22.0)	7062 (62.4)	9096 (90.4)	
Current	1505 (74.9)	3576 (31.6)	463 (4.6)	
Former	63 (3.1)	672 (5.9)	500 (5.0)	
Smoking status, n (%)				<0.001
Never	697 (34.7)	7100 (63.0)	8751 (87.4)	
Current	1073 (54.3)	2791 (24.8)	398 (4.0)	
Former	241 (12.0)	1377 (12.2)	861 (8.6)	
Hypertension, n (%)	1382 (34.4)	4177 (31.8)	1609 (26.4)	<0.001
Hyperlipidemia, n (%)	928 (23.2)	2404 (18.3)	920 (15.1)	<0.001
Gallstones, n (%)	458 (11.4)	1504 (11.5)	679 (11.2)	<0.001
Diabetes, n (%)	323 (8.1)	1198 (9.1)	434 (7.1)	<0.001
CVD, n (%)	686 (17.1)	2057 (15.7)	740 (12.2)	<0.001
Vegetables and fruits (both more than daily), n (%)	1248 (30.9)	6254 (47.2)	4689 (76.4)	<0.001
Meat (less than daily), n (%)	2320 (57.5)	8593 (64.9)	5112 (83.3)	<0.001
Systolic pressure, mm Hg	131.1 (20.0)	129.5 (19.7)	128.1 (19.3)	<0.001
Diastolic pressure, mm Hg	79.1 (11.8)	77.7 (11.3)	76.6 (10.7)	<0.001
Fasting glucose, mean (SD), mmol/L	5.8 (1.5)	5.8 (1.4)	5.7 (1.3)	<0.001
Total cholesterol, mmol/L	4.9 (1.0)	5.0 (1.0)	5.1 (1.0)	<0.001
Triglycerides, mmol/L	1.4 (1.0)	1.3 (0.9)	1.3 (0.9)	<0.001
HDL-C, mmol/L	1.5 (0.4)	1.5 (0.4)	1.5 (0.4)	<0.001
LDL-C, mmol/L	2.8 (0.8)	2.9 (0.8)	2.9 (0.8)	<0.001
Alkaline phosphatase, mmol/L	87.4 (26.0)	89.0 (30.0)	90.7 (34.1)	<0.001
γ-glutamyl transpeptidase, mmol/L	29.3 (35.2)	24.1 (28.1)	20.9 (19.0)	<0.001
AST, mmol/L	24.6 (14.2)	24.5 (14.4)	24.1 (10.7)	0.085
ALT, mmol/L	22.2 (16.6)	21.9 (19.8)	21.1 (14.3)	0.005
Physical activity (h/wk)	7.0 (6.4)	8.7 (7.8)	10.2 (7.5)	<0.001
Sleep duration (h/d)	8.5 (1.2)	8.2 (1.1)	7.9 (0.9)	<0.001

Continuous variables were displayed as means and standard deviation and categorical variables were expressed as numbers and percentages. Analysis of variance for continuous variables and χ^2^ test for categoric variables. Abbreviation: BMI, body mass index; CVD, cardiovascular disease; HDL-C, high-density lipoprotein cholesterol; LDL-C, low-density lipoprotein cholesterol; AST, aspartate transaminase; ALT, alanine aminotransferase.

**Table 2:** Associations of healthy lifestyle score with MAFLD and specific subtypes.

Characteristics	Lifestyle Score		
	Poor (0–2)	Intermediate (3–4)	Ideal (5–6)
Overall			
No. of cases	2248	6617	2627
Univariate model	1 [Reference]	0.80 (0.74–0.85)	0.60 (0.55–0.65)
Model ^a^	1 [Reference]	0.83 (0.77–0.89)	0.65 (0.60–0.70)
Model ^b^	1 [Reference]	0.84 (0.78–0.90)	0.68 (0.62–0.74)
Model ^c^	1 [Reference]	0.79 (0.73–0.86)	0.62 (0.57–0.68)
MAFLD with excess weight or obesity			
No. of cases	1869	4614	1228
Univariate model	1 [Reference]	0.62 (0.58–0.67)	0.29 (0.26–0.31)
Model ^a^	1 [Reference]	0.64 (0.60–0.69)	0.31 (0.28–0.34)
Model ^b^	1 [Reference]	0.66 (0.61–0.71)	0.33 (0.30–0.36)
Model ^c^	1 [Reference]	0.63 (0.58–0.68)	0.31 (0.28–0.34)
MAFLD with diabetes			
No. of cases	212	759	269
Univariate model	1 [Reference]	1.10 (0.94–1.28)	0.83 (0.69–0.99)
Model ^a^	1 [Reference]	1.10 (0.94–1.28)	0.83 (0.69–1.00)
Model ^b^	1 [Reference]	1.09 (0.89–1.34)	1.04 (0.82–1.33)
Model ^c^	1 [Reference]	1.05 (0.85–1.31)	0.97 (0.75–1.26)
MAFLD with MD			
No. of cases	2148	6209	2344
Univariate model	1 [Reference]	0.75 (0.70–0.80)	0.52 (0.48–0.57)
Model ^a^	1 [Reference]	0.79 (0.74–0.85)	0.54 (0.58–0.63)
Model ^b^	1 [Reference]	0.80 (0.75–0.87)	0.61 (0.56–0.67)
Model ^c^	1 [Reference]	0.76 (0.70–0.82)	0.56 (0.51–0.62)

Data are presented as ORs (95% CI). ^a^ Adjusted for age (continuous), sex (male vs. female), and education (less than high school, high school or equivalent, college or above). ^b^ Further adjusted for hypertension (yes or no), CVD (yes or no), hyperlipidemia (yes or no), and diabetes (yes or no). ^c^ Further adjusted for total cholesterol (continuous) and LDL-C (continuous). Abbreviation: MAFLD, metabolic dysfunction-associated fatty liver disease; MD, metabolic dysregulation; CI, confidence interval.

**Table 3:** Associations of lifestyle changes with risks of MAFLD and specific subtypes.

Measures	Change in Lifestyle Score, ORs (95% CIs)			
	Consistently Low	High to Low	Low to High	Consistently High
No. of cases	666	594	133	34
MAFLD	1 [Reference]	0.88 (0.74–1.04)	0.76 (0.68–0.86)	0.71 (0.61–0.82)
MAFLD with excess weight or obesity	1 [Reference]	0.85 (0.71–1.00)	0.72 (0.63–0.81)	0.64 (0.56–0.74)
MAFLD with diabetes	1 [Reference]	0.76 (0.47–1.25)	0.74 (0.54–1.02)	0.87 (0.59–1.28)
MAFLD with MD	1 [Reference]	0.79 (0.66–0.94)	0.49 (0.43–0.55)	0.41 (0.35–0.48)

Data are presented as ORs (95% CIs). Adjusted for age (continuous), sex (male vs. female) and education (less than high school, high school or equivalent, college or above), hypertension (yes or no), CVD (yes or no), hyperlipidemia (yes or no), diabetes (yes or no), total cholesterol (continuous), and LDL-C (continuous). Abbreviation: MAFLD, metabolic dysfunction-associated fatty liver disease; MD, metabolic dysregulation; CI, confidence interval.

## Data Availability

The datasets used and/or analyzed in the current study are available from the corresponding author upon reasonable request.

## Supplementary Materials

The following supporting information can be downloaded at: https://www.mdpi.com/article/10.3390/nu15214588/s1. Table S1: The definition and scoring of healthy and unhealthy lifestyle factors. Table S2: The diagnostic criteria of MAFLD in Asians. Table S3: Baseline characteristics for participants included and excluded due to missing data on the six lifestyle factors. Table S4: The participants’ lab test results at baseline and the end of follow-up. Table S5: Associations of weighted healthy lifestyle score with MAFLD and specific subtypes. Table S6: Associations of weighted healthy lifestyle score with MAFLD in individuals stratified by different sociodemographic and common comorbidities. Table S7: Associations between weighted healthy lifestyle score and MAFLD with excess weight or obesity in individuals stratified by different demographic and common comorbidities. Table S8: Associations of weighted healthy lifestyle score with incident MAFLD in individuals with diabetes stratified by different demographic and common comorbidities. Table S9: Associations of weighted healthy lifestyle score with MAFLD in individuals with metabolic dysregulation stratified by different demographic and common comorbidities. Table S10: Associations of each healthy lifestyle factor with risks of MAFLD and specific subtypes. Table S11: ORs (95% CIs) for one score increase according to three basic lifestyle factors. Table S12: Associations of five different baseline lifestyle scores with MAFLD and specific subtypes. Table S13: Associations of healthy lifestyle score with MAFLD and specific subtypes after excluding participants with CVD at baseline. Table S14: Associations of healthy lifestyle score with MAFLD and specific subtypes after excluding participants with incomplete information at baseline. Table S15: Associations of healthy lifestyle score with MAFLD and specific subtypes after mental health adjustment. Table S16: Associations of healthy lifestyle score with MAFLD and specific subtypes after using WHO BMI cutoff. Table S17: Associations of healthy lifestyle score with MAFLD and specific subtypes after using multiple imputations. Figure S1: Flowchart. Figure S2: Multivariate-adjusted spline curves for associations of weighted lifestyle score with the risks of MAFLD and specific subtypes. Figure S3: Stratified analysis of association of weighted healthy lifestyle score with an incident of MAFLD and its specific subtypes. References [33,34,35,36,37] cited in Supplementary Materials.
