# Supplementary material for: Associations of Combined Lifestyle Factors with MAFLD and the Specific Subtypes in Middle-Aged and Elderly Adults: The Dongfeng-Tongji Cohort Study
Source: Nutrients. 2023 Oct 28;15(21):4588. doi: 10.3390/nu15214588 (PMC10650607; doi:10.3390/nu15214588)
Supplement: Supplementary file 1 [file nutrients-15-04588-s001.zip › nutrients-2621665-supplementary.pdf]

Supplementary Materials

Associations of Combined Lifestyle Factors with MAFLD and the Specific Subtypes in Middle-Aged and Elderly Adults: The Dongfeng-Tongji Cohort Study

Hongxia Li [1,2,†], Zhiqiang Cao [1,2,†], Jingxi Li [1,2], Lei King [1,2], Zhuangyu Zhang [1,2], Ying Zhao [1,2], Siyi Zhang [1,2], Yajing Song [1,2], Qian Zhang [1,2], Liangkai Chen [1,2], Yuhan Tang [1,2], Lingling Dai [3,*] and Ping Yao [1,2,*]

† These authors contributed equally to this work.

Corresponding authors: Ping Yao (yaoping@mails.tjmu.edu.cn) and Lingling Dai (daily@hust.edu.cn)

Supplementary Table S1 The definition and scoring of healthy and unhealthy lifestyle factors

Supplementary Table S2 The diagnostic criteria of MAFLD in Asians

Supplementary Table S3 Baseline characteristics for participants included and excluded due to missing data on the six lifestyle factors

Supplementary Table S4 The participants' lab test results at baseline and the end of follow-up

Supplementary Table S5 Associations of weighted healthy lifestyle score with MAFLD and specific subtypes

Supplementary Table S6 Associations of weighted healthy lifestyle score with MAFLD in individuals stratified by different sociodemographic and common comorbidities

Supplementary Table S7 Associations between weighted healthy lifestyle score and MAFLD with overweight or obesity in individuals stratified by different demographic and common comorbidities

Supplementary Table S8 Associations of weighted healthy lifestyle score with incident MAFLD in individuals with diabetes stratified by different demographic and

common comorbidities

Supplementary Table S9 Associations of weighted healthy lifestyle score with MAFLD in individuals with metabolic dysregulation stratified by different demographic and common comorbidities

Supplementary Table S10 Associations of each healthy lifestyle factor with risks of MAFLD and specific subtypes

Supplementary Table S11 ORs (95% CIs) for one score increase according to three basic lifestyle factors

Supplementary Table S12 Associations of five different baseline lifestyle scores with MAFLD and specific subtypes

Supplementary Table S13 Associations of healthy lifestyle score with MAFLD and specific subtypes after excluding participants with CVD at baseline

Supplementary Table S14 Associations of healthy lifestyle score with MAFLD and specific subtypes after excluding participants with incomplete information at baseline

Supplementary Table S15 Associations of healthy lifestyle score with MAFLD and specific subtypes after mental health adjustment

Supplementary Table S16 Associations of healthy lifestyle score with MAFLD and specific subtypes after using WHO BMI cutoff

Supplementary Table S17 Associations of healthy lifestyle score with MAFLD and specific subtypes after using multiple imputations

Supplementary Figure S1 Flowchart

Supplementary Figure S2 Multivariate-adjusted spline curves for associations of weighted lifestyle score with the risks of MAFLD and specific subtypes

Supplementary Figure S3 Stratified Analysis of association of weighted healthy lifestyle score with an incident of MAFLD and its specific subtypes

Methods

Study population

The Dongfeng-Tongji (DFTJ) cohort study is a dynamic cohort, and detailed information on this cohort has been described elsewhere[1]. DFTJ was launched in 2008, and 27009 retired employees from Dongfeng Motor Corporation were recruited and completed baseline questionnaires and medical examinations between September 2008 and June 2010. As shown in Supplementary Figure 1, 12829 participants (n=8907 in 2008-2010, n=3922 in 2013) with prevalent MAFLD were excluded at baseline and 1719 participants (n=1026 in 2008-2010, n=693 in 2013) were lost to follow-up. Additionally, 1061 participants (n=841 in 2008-2010, n=220 in 2013) with cancer and 2112 participants with missing information on lifestyle (n=1589 in 2008-2010, n=523 in 2013). Finally, a total of 23408 participants were included in the present study.

In this study, all research was conducted following both the Declarations of Helsinki and Istanbul; all research was approved by the appropriate ethics and institutional review committees; all participants provided written informed consent and our study was approved by the Ethics and Human Subject Committee of Tongji Medical College, Huazhong University of Science and Technology.

Definition of lifestyle factors and scores

Baseline lifestyle factors contain body mass index (BMI), tobacco smoking status, drinking status, diet, physical activity, and sleep. Tobacco smoking status included the beginning and quitting age and the amount of tobacco smoked/day. Although several studies displayed that low to moderate levels of alcohol intake may have a protective effect on NAFLD[2], however, a study published in *Lancet* carried out in 195 countries and territories and including 650000 individuals found the safest level of drinking is none[3], so we defined participants who reported never drinking alcohol as being at low risk for alcohol consumption status in our present study. Drinking status included the beginning, drinking frequency, type of alcoholic beverage, volume of alcoholic

beverage/time, and quitting age. Information about physical activity included the frequency per week, the type, and the mean duration of leisure-time physical activity in the past six months. Total duration per week was calculated as duration (hours per time) × frequency (times per week). Exercise types included walking, biking, dancing, tai chi, jogging, swimming, climbing stairs/ mountains, and playing basketball/volleyball/ soccer or others. Information on diet was collected by a simplified semiquantitative food frequency questionnaire which included several conventional food groups in the past 12 months. Weight, height, and circumference were obtained in light indoors and without shoes. BMI is an important factor reflecting whether the lifestyle is in ideal condition, and it's a pivotal index to predict the risk of MAFLD[4]. BMI was calculated as weight in kilograms divided by height in meters squared. Sleep is an indispensable element of human biology and a requirement for life, and it is nearly involved in every physiological system. Therefore, the American Heart Association (AHA) was elected to add sleep duration as an eighth metric to be Life's Essential 8[5]. Considering the J-shaped association between sleep duration and the risk of MAFLD, nighttime sleep duration was grouped into optimal (7-9 hours/day) and not optimal (<7 or >9 hours/day). Other information such as age, sex, education (primary school/middle school/high school/college or more), and mental status were also included in this questionnaire.

Assessment of other variables or covariates

In our study, other covariates, including age, sex, education attainment, hypertension, hyperlipidemia, CVD (including stroke, coronary heart disease, myocardial infarction), diabetes, and mental stress (available only in the first survey) were obtained by the questionnaires or clinical examination. Briefly, anthropometric indices, systolic blood pressure (SBP), diastolic blood pressure (DBP), and waist circumference (WC) were

measured by trained investigators. Metabolic biomarkers, including fasting glucose, total cholesterol (TC), low-density lipoprotein cholesterol (LDL-C), γ-glutamyl transpeptidase, alkaline phosphatase, aspartate aminotransferase (AST), alanine transaminase (ALT), and C-reactive protein were measured among the participants who provided blood samples at baseline and follow-up surveys. Gallstone was dichotomized as yes or no which was assessed by B ultrasound. The detailed information is documented elsewhere[6].

Statistical analysis

The univariate and multivariable-adjusted odds ratios (ORs) with a 95% confidence interval (CI) of lifestyle for MAFLD were examined using logistic regression analysis. Lifestyle score was categorized into poor, intermediate, and ideal groups; the reference group was the poor lifestyle category. In model[a], we adjusted for age (continuous), sex (male or female), and education attainment (less than high school, high school or equivalent, or college or above). In model[b], we further adjusted for hypertension status (yes or no), hyperlipidemia (yes or no), CVD (yes or no), and diabetes (yes or no). In model[c], we additionally adjusted for LDL-C (continuous) and TC (continuous). To assess the association of lifestyle changes with the risk of MAFLD, we executed analysis in 10960 participants who completed baseline and subsequent follow-up surveys in 2013. Lifestyle changes were defined as the changes from the baseline to the first follow-up, due to samples divided into nine groups (poor to the poor group, intermediate to the poor group, ideal to the poor group, poor to intermediate group, intermediate to intermediate group, ideal to intermediate group, poor to the ideal group, intermediate to the ideal group, ideal to ideal group, respectively) were very uneven, we combined the poor group and intermediate group as the low-scoring group for subsequent analyses, which was categorized participants into 4 groups: consistently low, high to low, low to high, consistently high, and set the consistently low as the reference. The important covariates were the stratified factors. Potential interaction between lifestyle and stratification factors was evaluated by introducing a multiplicative term between lifestyle and stratification variables as continuous variables into the multivariate models, and false discovery rate (FDR) was used to control a relatively

low proportion of false positives. A restricted cubic spline model with 3 knots (10th, 50th, 90th) was utilized to test the dose-response association between lifestyle score and incident MAFLD or specific subtypes among the elderly participants.

The important covariates were the stratified factors. Potential interaction between lifestyle and stratification factors was evaluated by introducing a multiplicative term between lifestyle and stratification variables as continuous variables into the multivariate models, and testing whether the coefficient of the interaction term was equal to zero. Taking increased false positives in multiple hypothesis testing into account, we adjusted the *P* value with Bonferroni correction. We also calculated the false discovery rate (FDR) to identify as many significant interactions as possible while controlling a relatively low proportion of false positives, and FDR <0.05 was considered significant. Moreover, several secondary analyses were conducted. Firstly, evaluating multiple lifestyle factors with equal weight might limit us to concluding individual risk factors, and lifestyle score calculated according to the actual weight of each lifestyle factor is more appropriate[7]. Therefore, weighted lifestyle scores based on β coefficients of each lifestyle factor in the logistic regression model were conducted to highlight the more important factors in this study[8]. Secondly, stratified analyses were conducted by age (<65 or ≥ 65 years), sex (male or female), hypertension (yes or no), and hyperlipidemia (yes or no), weighted lifestyle score was included in models as categorical. Thirdly, we further assessed the association of different 5 lifestyle factors with outcomes by removing one lifestyle factor each time. Fourthly, we assessed the association of different components of lifestyle factors by including one lifestyle factor each time based on three lifestyle factors (BMI, smoking, alcohol consumption).

Furthermore, we performed several sensitivity analyses. First of all, after the diagnosis of CVD, participants might change their lifestyle, to reduce possible confounding, we excluded participants with prevalent CVD. Second, we redefined the healthy level of BMI with the criterion of the World Health Organization (WHO). Third, given the potential confounding of psychological factors in association with lifestyle with MAFLD, we further adjusted the mental stress factor in a subset of this study population. Fourth, to exclude the effects of incomplete data, participants with complete data were

included for analysis. Finally, we also input missing covariates by multiple imputations to test the association of lifestyle with MAFLD and the specific subtypes.

Firstly, evaluating multiple lifestyle factors with equal weight might limit us to concluding individual risk factors, and lifestyle score calculated according to the actual weight of each lifestyle factor is more appropriate[7]. Therefore, weighted lifestyle scores based on β coefficients of each lifestyle factor in the logistic regression model were conducted to highlight the more important factors in this study[8]. Secondly, stratified analyses were conducted by age (<65 or ≥ 65 years), sex (male or female), hypertension (yes or no), and hyperlipidemia (yes or no), weighted lifestyle score was included in models as categorical. Thirdly, we further assessed the association of different 5 lifestyle factors with outcomes by removing one lifestyle factor each time. Fourthly, we assessed the association of different components of lifestyle factors by including one lifestyle factor each time based on three lifestyle factors (BMI, smoking, alcohol consumption).

Furthermore, we performed several sensitivity analyses. First of all, after the diagnosis of CVD, participants might change their lifestyle, to reduce possible confounding, we excluded participants with prevalent CVD. Second, we redefined the healthy level of BMI with the criterion of the World Health Organization (WHO). Third, given the potential confounding of psychological factors in association with lifestyle with MAFLD, we further adjusted the mental stress factor in a subset of this study population. Fourth, to exclude the effects of incomplete data, participants with complete data were included for analysis. Finally, we also input missing covariates by multiple imputations to test the association of lifestyle with MAFLD and the specific subtypes.

Supplementary Table S1 The definition and scoring of healthy and unhealthy lifestyle factors

Factor	Healthy (1 point)	Unhealthy (0 point)
Smoking	Never smoking	Former smoking or current smoking
Drinking	Never drinking	Former drinking or current drinking
Diet	consuming vegetables and fruit daily and not consuming meat daily	not consuming vegetables or fruit daily or consuming meat daily
BMI	BMI of 18.5 to 23.9 kg/m^2	BMI of 18.5 or ≥24 kg/m^2
Physical activity	≥150 min/week	<150 min/week

Supplementary Table S2 The diagnostic criteria of MAFLD in Asians

Definition	Diagnostic criteria
MAFLD	**The presence of hepatic steatosis in adults (examined by B ultrasound)**<br>(With the presence of at least one of the following 3 metabolic conditions)
	**Condition 1: BMI ≥23 kg/m^2 in Asians**
	**Condition 2: Type 2 diabetes mellitus**
	**Condition 3: at least two metabolic dysregulations**<br>• Waist circumference ≥90/80 cm in Asian men and women<br>• Blood pressure ≥130/85 mmHg or treatment with antihypertensive drugs<br>• Plasma triglycerides ≥1.70 mmol/L (150 mg/dL) or treatment with lipid-lowering drugs<br>• Plasma high-density lipoprotein cholesterol <1.0 mmol/L (40 mg/dL) for men and <1.3 mmol/L (50 mg/dL) for women or specific drug treatment<br>• Prediabetes: fasting glucose levels 5.6 to 6.9 mmol/L<br>• Homeostasis model assessment-insulin resistance score (HOMA-IR) ≥2.5*<br>• Plasma high-sensitivity C-reactive protein concentration >2 mg/L

* HOMA-IR is not available in this study

Supplementary Table S3 Baseline characteristics for participants included and excluded due to missing data on the six lifestyle factors

Characteristics	Include	Exclude	*P*-value
N	23408	2112	
Age, y	61.7 (8.4)	61.0 (8.0)	< 0.001
BMI, kg/m^2	23.3 (2.8)	24.4 (3.5)	< 0.001
Waist, cm	80.5 (8.4)	83.0 (9.5)	< 0.001
Male, n (%)	10408 (44.5)	1727 (39.7)	< 0.001
Education attainment, n (%)			< 0.001
Less than high school	13424 (57.7)	2747 (63.8)	
High school or equivalent	6948 (29.9)	1138 (26.4)	
College or above	2880 (12.4)	421 (9.8)	
Alcohol consumption, n (%)			< 0.001
Never	16629 (71.0)	3183 (75.0)	
Current	5544 (23.7)	899 (21.2)	
Former	1235 (5.3)	160 (3.8)	
Smoking status, n (%)			< 0.001
Never	16667 (71.2)	3023 (71.2)	
Current	4262 (18.2)	914 (21.5)	
Former	2479 (10.6)	307 (7.2)	
Hypertension, n (%)	7168 (30.8)	1374 (32.1)	< 0.001
Hyperlipidemia, n (%)	4252 (18.3)	801 (18.7)	< 0.001
Gall-stone, n (%)	2641 (11.4)	465 (10.9)	< 0.001
Diabetes, n (%)	1955 (8.4)	374 (8.8)	< 0.001
CVD, n (%)	3483 (15.0)	676 (15.8)	0.186
Vegetables and fruits (both more than daily), n (%)	112191 (52.1)	3408 (77.4)	< 0.001
Meat (less than daily), n (%)	7383 (31.5)	1243 (34.6)	< 0.001
Systolic pressure, mm Hg	129.4 (19.7)	129.7 (19.7)	0.468

Diastolic pressure, mm Hg	77.6 (11.2)	78.6 (11.5)	< 0.001
Fasting glucose, mmol/L, mmol/L	5.8 (1.4)	6.0 (1.7)	< 0.001
Total cholesterol, mmol/L	5.0 (1.0)	5.1 (1.0)	< 0.001
Total cholesterol, mmol/L	1.3 (0.9)	1.5 (1.3)	< 0.001
HDL, mmol/L	1.5 (0.4)	1.5 (0.4)	0.001
LDL-C, mmol/L	2.9 (0.8)	3.0 (0.8)	< 0.001
Alkaline phosphatase, mmol/L	98.1 (30.3)	92.0 (45.9)	< 0.001
γ-glutamyl transpeptidase, mmol/L	24.5 (28.2)	25.1 (24.7)	0.234
AST, mmol/L	24.4 (13.5)	24.8 (13.3)	0.147
ALT, mmol/L	21.8 (17.9)	23.5 (17.7)	< 0.001
Physical activity (h/wk)	8.9 (7.6)	9.5 (8.5)	< 0.001
Sleep duration (h/d)	8.1 (1.0)	8.2 (1.1)	< 0.001

Abbreviation: CVD, cardiovascular disease; HDL, high-density lipoprotein; Data are presented as mean (SD) for continuous variables or n (%) for categorical variables.

Supplementary Table S4 The participants' lab test results at baseline and the end of follow-up

Characteristics	Baseline	The end of follow-up
Systolic pressure, mean (SD), mm Hg	129.4 (19.7)	137.2 (23.2)
Diastolic pressure, mean (SD), mm Hg	77.6 (11.2)	78.1 (12.3)
Fasting glucose, mean (SD), mmol/L	5.8 (1.6)	5.8 (1.5)
Total cholesterol, mean (SD), mmol/L	5.0 (1.6)	4.8 (1.1)
Triglycerides, mean (SD), mmol/L	1.3 (0.9)	1.4 (0.9)
HDL-C, mean (SD), mmol/L	1.5 (0.4)	1.5 (0.4)
LDL-C, mean (SD), mmol/L	2.9 (0.8)	2.7 (0.9)
Alkaline phosphatase, mean (SD), mmol/L	89.1 (30.2)	88.5 (29.0)
γ-glutamyl transpeptidase, mean (SD), mmol/L	24.5 (28.1)	25.5 (33.1)
AST, mean (SD), mmol/L	24.4 (13.5)	23.1 (19.2)
ALT, mean (SD), mmol/L	21.7 (17.9)	22.6 (21.4)

Note: HDL-C, high-density lipoprotein cholesterol; LDL-C, low-density lipoprotein cholesterol; AST, aspartate transaminase; ALT, alanine transaminase.

Supplementary Table S5 Associations of weighted healthy lifestyle score with MAFLD and specific subtypes

Outcome	Lifestyle			Continuous[d]	*P* for trend
	First tertile (Poor)	Second tertile (Intermediate)	Third tertile (Ideal)		
Overall					
Univariate model	1 [Reference]	0.65 (0.59-0.71)	0.46 (0.43-0.49)	0.68 (0.66-0.70)	< 0.001
Model[a]	1 [Reference]	0.62 (0.57-0.68)	0.47 (0.44-0.49)	0.85 (0.80-0.90)	< 0.001
Model[b]	1 [Reference]	0.64 (0.58-0.70)	0.48 (0.46-0.51)	0.70 (0.68-0.72)	< 0.001
Model[c]	1 [Reference]	0.59 (0.53-0.65)	0.44 (0.41-0.46)	0.66 (0.64-0.68)	< 0.001
MAFLD with overweight or obesity					
Univariate model	1 [Reference]	0.30 (0.27-0.33)	0.11 (0.10-0.12)	0.33 (0.32-0.34)	< 0.001
Model[a]	1 [Reference]	0.28 (0.26-0.31)	0.11 (0.11-0.12)	0.33 (0.32-0.35)	< 0.001
Model[b]	1 [Reference]	0.29 (0.26-0.32)	0.12 (0.11-0.13)	0.34 (0.33-0.35)	< 0.001
Model[c]	1 [Reference]	0.27 (0.24-0.30)	0.11 (0.10-0.12)	0.32 (0.31-0.34)	< 0.001
MAFLD with diabetes					
Univariate model	1 [Reference]	0.70 (0.56-0.86)	0.67 (0.59-0.75)	0.81 (0.77-0.85)	< 0.001
Model[a]	1 [Reference]	0.72 (0.60-0.87)	0.72 (0.65-0.81)	0.85 (0.80-0.90)	< 0.001
Model[b]	1 [Reference]	0.98 (0.76-1.28)	0.80 (0.68-0.94)	0.90 (0.83-0.97)	< 0.001
Model[c]	1 [Reference]	0.93 (0.71-1.23)	0.75 (0.63-0.88)	0.86 (0.79-0.94)	< 0.001
MAFLD with MD					
Univariate model	1 [Reference]	0.62 (0.57-0.68)	0.41 (0.39-0.44)	0.64 (0.63-0.66)	< 0.001
Model[a]	1 [Reference]	0.59 (0.54-0.65)	0.43 (0.40-0.45)	0.65 (0.64-0.67)	< 0.001
Model[b]	1 [Reference]	0.61 (0.55-0.67)	0.45 (0.42-0.47)	0.67 (0.65-0.69)	< 0.001
Model[c]	1 [Reference]	0.56 (0.51-0.62)	0.40 (0.38-0.43)	0.63 (0.62-0.65)	< 0.001

Data are presented as odds ratios (95% CIs).

[a] Adjusted for age (continuous), sex (male or female), and education (less than high school, high school or equivalent, college or above).

[b] Further adjusted for hypertension (yes or no), CVD (yes or no), hyperlipidemia (yes or no), and diabetes (yes or no).

[c] Further adjusted for total cholesterol (continuous), and LDL-C (continuous).

[d] The associations were assessed with a weighted lifestyle score as a continuous variable.

Abbreviation: MAFLD, metabolic dysfunction-associated fatty liver disease; MD, metabolic dysregulation; CIs, confidence intervals.

Supplementary Table S6 Associations of weighted healthy lifestyle score with MAFLD in individuals stratified by different sociodemographic and common comorbidities

Outcome	Lifestyle			*P* for trend
	First tertile (Poor)	Second tertile (Intermediate)	Third tertile (Ideal)	
Age < 65				
Univariate model	1 [Reference]	0.63 (0.57-0.71)	0.43 (0.40-0.46)	< 0.001
Model[a]	1 [Reference]	0.61 (0.55-0.69)	0.45 (0.42-0.48)	< 0.001
Model[b]	1 [Reference]	0.63 (0.56-0.71)	0.46 (0.43-0.49)	< 0.001
Model[c]	1 [Reference]	0.58 (0.51-0.65)	0.40 (0.37-0.43)	< 0.001
Age ≥65				
Univariate model	1 [Reference]	0.68 (0.58-0.79)	0.50 (0.46-0.56)	< 0.001
Model[a]	1 [Reference]	0.65 (0.55-0.76)	0.52 (0.47-0.57)	< 0.001
Model[b]	1 [Reference]	0.68 (0.58-0.80)	0.53 (0.48-0.59)	< 0.001
Model[c]	1 [Reference]	0.64 (0.54-0.76)	0.52 (0.46-0.57)	< 0.001
Male				
Univariate model	1 [Reference]	0.56 (0.50-0.63)	0.44 (0.41-0.48)	< 0.001
Model[a]	1 [Reference]	0.55 (0.49-0.62)	0.44 (0.41-0.48)	< 0.001
Model[b]	1 [Reference]	0.57 (0.51-0.64)	0.46 (0.42-0.50)	< 0.001
Model[c]	1 [Reference]	0.51 (0.45-0.58)	0.41 (0.37-0.45)	< 0.001
Female				
Univariate model	1 [Reference]	0.78 (0.67-0.90)	0.49 (0.46-0.53)	< 0.001
Model[a]	1 [Reference]	0.76 (0.66-0.89)	0.49 (0.46-0.53)	< 0.001
Model[b]	1 [Reference]	0.78 (0.67-0.91)	0.51 (0.47-0.55)	< 0.001
Model[c]	1 [Reference]	0.75 (0.64-0.89)	0.46 (0.42-0.50)	< 0.001
hyperlipidemia				
Univariate model	1 [Reference]	0.70 (0.56-0.88)	0.50 (0.44 0.56)	< 0.001
Model[a]	1 [Reference]	0.68 (0.54-0.85)	0.53 (0.46-0.60)	< 0.001
Model[b]	1 [Reference]	0.69 (0.55-0.86)	0.54 (0.47-0.62)	< 0.001

Model[c]	1 [Reference]	0.68 (0.54-0.87)	0.50 (0.43-0.58)	< 0.001
Not hyperlipidemia				
Univariate model	1 [Reference]	0.65 (0.59-0.72)	0.46 (0.44-0.49)	< 0.001
Model[a]	1 [Reference]	0.65 (0.58-0.72)	0.46 (0.43-0.49)	< 0.001
Model[b]	1 [Reference]	0.63 (0.57-0.70)	0.47 (0.44-0.50)	< 0.001
Model[c]	1 [Reference]	0.57 (0.51-0.64)	0.42 (0.39-0.45)	< 0.001
hypertension				
Univariate model	1 [Reference]	0.83 (0.70-0.98)	0.52 (0.47-0.58)	< 0.001
Model[a]	1 [Reference]	0.79 (0.67-0.94)	0.53 (0.48-0.59)	< 0.001
Model[b]	1 [Reference]	0.81 (0.68-0.96)	0.55 (0.49-0.61)	< 0.001
Model[c]	1 [Reference]	0.72 (0.60-0.87)	0.49 (0.44-0.54)	< 0.001
Not hypertension				
Univariate model	1 [Reference]	0.59 (0.53-0.66)	0.45 (0.42-0.48)	< 0.001
Model[a]	1 [Reference]	0.59 (0.53-0.66)	0.45 (0.42-0.48)	< 0.001
Model[b]	1 [Reference]	0.58 (0.52-0.65)	0.46 (0.43-0.49)	< 0.001
Model[c]	1 [Reference]	0.54 (0.48-0.61)	0.41 (0.38-0.44)	< 0.001

Demographic characteristics (age and sex) and common comorbidities (hypertension and hyperlipidemia) were stratified to analyze the association between MAFLD and combined lifestyle scores. Data are presented as odds ratios (95% CIs).

[a] Adjusted for age (continuous), sex (male or female), and education (less than high school, high school or equivalent, college or above).

[b] Further adjusted for hypertension (yes or no), CVD (yes or no), hyperlipidemia (yes or no), and diabetes (yes or no).

[c] Further adjusted for total cholesterol (continuous), and LDL-C (continuous).

Abbreviation: MAFLD, metabolic dysfunction-associated fatty liver disease; MD, metabolic dysregulation; CIs, confidence intervals.

Supplementary Table S7 Associations between weighted lifestyle score and MAFLD with overweight or obesity in individuals stratified by demographic and common comorbidities

Outcome	Lifestyle			*P* for trend
	First tertile (Poor)	Second tertile (Intermediate)	Third tertile (Ideal)	
Age < 65				
Univariate model	1 [Reference]	0.29 (0.26-0.33)	0.10 (0.09-0.11)	< 0.001
Model[a]	1 [Reference]	0.28 (0.25-0.32)	0.11 (0.10-0.12)	< 0.001
Model[b]	1 [Reference]	0.29 (0.25-0.32)	0.11 (0.10-0.12)	< 0.001
Model[c]	1 [Reference]	0.26 (0.23-0.30)	0.10 (0.09-0.11)	< 0.001
Age≥65				
Univariate model	1 [Reference]	0.31 (0.26-0.37)	0.13 (0.11-0.15)	< 0.001
Model[a]	1 [Reference]	0.30 (0.25-0.36)	0.13 (0.12-0.15)	< 0.001
Model[b]	1 [Reference]	0.31 (0.26-0.37)	0.14 (0.12-0.15)	< 0.001
Model[c]	1 [Reference]	0.31 (0.25-0.37)	0.13 (0.12-0.15)	< 0.001
Male				
Univariate model	1 [Reference]	0.21 (0.19-0.24)	0.12 (0.11-0.13)	< 0.001
Model[a]	1 [Reference]	0.21 (0.18-0.24)	0.12 (0.11-0.13)	< 0.001
Model[b]	1 [Reference]	0.22 (0.19-0.25)	0.12 (0.11-0.14)	< 0.001
Model[c]	1 [Reference]	0.20 (0.17-0.23)	0.11 (0.10-0.12)	< 0.001
Female				
Univariate model	1 [Reference]	0.48 (0.41-0.56)	0.11 (0.10-0.12)	< 0.001
Model[a]	1 [Reference]	0.47 (0.40-0.55)	0.11 (0.10-0.12)	< 0.001
Model[b]	1 [Reference]	0.47 (0.40-0.55)	0.12 (0.11-0.13)	< 0.001
Model[c]	1 [Reference]	0.46 (0.38-0.54)	0.11 (0.10-0.12)	< 0.001
Hypertension				
Univariate model	1 [Reference]	0.44 (0.37-0.52)	0.14 (0.12-0.15)	< 0.001
Model[a]	1 [Reference]	0.42 (0.35-0.50)	0.14 (0.12-0.16)	< 0.001
Model[b]	1 [Reference]	0.42 (0.36-0.50)	0.14 (0.12-0.16)	< 0.001

Model[c]	1 [Reference]	0.38 (0.32-0.46)	0.12 (0.11-0.14)	< 0.001
Not hypertension				
Univariate model	1 [Reference]	0.25 (0.22-0.28)	0.11 (0.10-0.11)	< 0.001
Model[a]	1 [Reference]	0.25 (0.22-0.28)	0.10 (0.10-0.11)	< 0.001
Model[b]	1 [Reference]	0.24 (0.21-0.27)	0.11 (0.10-0.12)	< 0.001
Model[c]	1 [Reference]	0.23 (0.20-0.26)	0.10 (0.09-0.11)	< 0.001
Hyperlipidemia				
Univariate model	1 [Reference]	0.40 (0.32-0.50)	0.12 (0.10-0.14)	< 0.001
Model[a]	1 [Reference]	0.39 (0.31-0.48)	0.13 (0.11-0.15)	< 0.001
Model[b]	1 [Reference]	0.39 (0.31-0.49)	0.13 (0.11-0.15)	< 0.001
Model[c]	1 [Reference]	0.39 (0.30-0.49)	0.13 (0.11-0.15)	< 0.001
Not hyperlipidemia				
Univariate model	1 [Reference]	0.28 (0.25-0.32)	0.11 (0.10-0.12)	< 0.001
Model[a]	1 [Reference]	0.28 (0.25-0.31)	0.11 (0.10-0.12)	< 0.001
Model[b]	1 [Reference]	0.27 (0.24-0.30)	0.11 (0.11-0.12)	< 0.001
Model[c]	1 [Reference]	0.25 (0.22-0.28)	0.10 (0.09-0.11)	< 0.001

Demographic characteristics (age and sex) and common comorbidities (hypertension and hyperlipidemia) were stratified to analyze the association between MAFLD with overweight or obesity and combined lifestyle scores. Data are presented as odds ratios (95% CIs).

[a] Adjusted for age (continuous), sex (male or female), and education (less than high school, high school or equivalent, college or above).

[b] Further adjusted for hypertension (yes or no), CVD (yes or no), hyperlipidemia (yes or no), and diabetes (yes or no).

[c] Further adjusted for total cholesterol (continuous) and LDL-C (continuous).

Abbreviation: MAFLD, metabolic dysfunction-associated fatty liver disease; MD, metabolic dysregulation; CIs, confidence intervals.

Supplementary Table S8 Associations of weighted lifestyle score with incident MAFLD in individuals with diabetes stratified by demographic and common comorbidities

Outcome	Lifestyle			*P* for trend
	First tertile (Poor)	Second tertile (Intermediate)	Third tertile (Ideal)	
Age < 65				
Univariate model	1 [Reference]	0.75 (0.59-0.96)	0.65 (0.56-0.76)	< 0.001
Model[a]	1 [Reference]	0.69 (0.54-0.89)	0.72 (0.62-0.83)	< 0.001
Model[b]	1 [Reference]	0.98 (0.69-1.40)	0.84 (0.68-1.04)	0.102
Model[c]	1 [Reference]	0.93 (0.64-1.35)	0.74 (0.59-0.94)	0.967
Age≥65				
Univariate model	1 [Reference]	0.76 (0.57-1.01)	0.72 (0.61-0.85)	< 0.001
Model[a]	1 [Reference]	0.74 (0.55-0.98)	0.72 (0.61-0.85)	< 0.001
Model[b]	1 [Reference]	1.01 (0.68-1.50)	0.76 (0.60-0.96)	0.022
Model[c]	1 [Reference]	0.94 (0.62-1.44)	0.75 (0.59-0.97)	0.027
Male				
Univariate model	1 [Reference]	0.65 (0.52-0.82)	0.73 (0.63-0.86)	< 0.001
Model[a]	1 [Reference]	0.66 (0.52-0.83)	0.74 (0.64-0.87)	< 0.001
Model[b]	1 [Reference]	0.93 (0.68-1.28)	0.71 (0.57-0.88)	0.002
Model[c]	1 [Reference]	0.85 (0.60-1.19)	0.63 (0.49-0.79)	< 0.001
Female				
Univariate model	1 [Reference]	0.82 (0.59-1.14)	0.66 (0.56-0.77)	< 0.001
Model[a]	1 [Reference]	0.97 (0.75-1.24)	0.77 (0.66-0.90)	< 0.001
Model[b]	1 [Reference]	1.20 (0.84-1.73)	0.81 (0.65-1.01)	0.434
Model[c]	1 [Reference]	1.13 (0.76-1.66)	0.74 (0.58-0.93)	0.440
Hypertension				
Univariate model	1 [Reference]	0.96 (0.75-1.24)	0.75 (0.64-0.88)	0.003
Model[a]	1 [Reference]	1.20 (0.98-1.60)	1.14 (0.81-1.59)	0.019
Model[b]	1 [Reference]	1.21 (0.92-1.53)	0.99 (0.75-1.29)	0.266

Model[c]	1 [Reference]	1.10 (0.81-1.48)	0.97 (0.67-1.40)	0.071
Not hypertension				
Univariate model	1 [Reference]	1.09 (0.86-1.40)	0.91 (0.69-1.20)	< 0.001
Model[a]	1 [Reference]	1.14 (0.92-1.41)	1.00 (0.78-1.29)	< 0.001
Model[b]	1 [Reference]	0.97 (0.72-1.30)	0.95 (0.67-1.33)	0.008
Model[c]	1 [Reference]	1.00 (0.73-1.37)	0.96 (0.66-1.37)	0.003
Hyperlipidemia				
Univariate model	1 [Reference]	0.90 (0.66-1.23)	0.75 (0.63-0.91)	< 0.001
Model[a]	1 [Reference]	0.89 (0.65-1.22)	0.80 (0.66-0.97)	0.002
Model[b]	1 [Reference]	1.15 (0.72-1.84)	0.85 (0.65-1.12)	0.079
Model[c]	1 [Reference]	1.04 (0.63-1.70)	0.76 (0.57-1.02)	0.014
Not hyperlipidemia				
Univariate model	1 [Reference]	0.76 (0.60 -0.96)	0.70 (0.61-0.80)	< 0.001
Model[a]	1 [Reference]	0.75 (0.59 -0.96)	0.75 (0.65-0.86)	0.005
Model[b]	1 [Reference]	0.91 (0.66-1.25)	0.77 (0.64-0.94)	0.032
Model[c]	1 [Reference]	0.89 (0.64-1.25)	0.74 (0.60-0.90)	0.017

Demographic characteristics (age and sex) and common comorbidities (hypertension and hyperlipidemia) were stratified to analyze the association between MAFLD with overweight or obesity and combined lifestyle scores. Data are presented as odds ratios (95% CIs).

[a] Adjusted for age (continuous), sex (male or female), and education (less than high school, high school or equivalent, college or above).

[b] Further adjusted for hypertension (yes or no), CVD (yes or no), hyperlipidemia (yes or no), and diabetes (yes or no).

[c] Further adjusted for total cholesterol (continuous) and LDL-C (continuous).

Abbreviation: MAFLD, metabolic dysfunction-associated fatty liver disease; MD, metabolic dysregulation; CIs, confidence intervals.

Supplementary Table S9 Associations of weighted lifestyle score with MAFLD in individuals with metabolic dysregulation stratified by demographic and common comorbidities

Outcome	Lifestyle			*P* for trend
	First tertile (Poor)	Second tertile (Intermediate)	Third tertile (Ideal)	
Age < 65				
Univariate model	1 [Reference]	0.60 (0.54-0.68)	0.39 (0.36-0.41)	< 0.001
Model[a]	1 [Reference]	0.58 (0.51-0.64)	0.40 (0.38-0.43)	< 0.001
Model[b]	1 [Reference]	0.59 (0.53-0.67)	0.42 (0.39-0.45)	< 0.001
Model[c]	1 [Reference]	0.54 (0.48-0.61)	0.37 (0.34-0.40)	< 0.001
Age≥65				
Univariate model	1 [Reference]	0.65 (0.55-0.76)	0.47 (0.42-0.52)	< 0.001
Model[a]	1 [Reference]	0.62 (0.53-0.72)	0.48 (0.44-0.53)	< 0.001
Model[b]	1 [Reference]	0.65 (0.55-0.76)	0.50 (0.45-0.55)	< 0.001
Model[c]	1 [Reference]	0.64 (0.52-0.73)	0.49 (0.44-0.54)	< 0.001
Male				
Univariate model	1 [Reference]	0.52 (0.46-0.59)	0.41 (0.37-0.44)	< 0.001
Model[a]	1 [Reference]	0.51 (0.46-0.58)	0.41 (0.37-0.44)	< 0.001
Model[b]	1 [Reference]	0.54 (0.48-0.61)	0.42 (0.39-0.46)	< 0.001
Model[c]	1 [Reference]	0.48 (0.42-0.55)	0.38 (0.34-0.42)	< 0.001
Female				
Univariate model	1 [Reference]	0.74 (0.64-0.86)	0.45 (0.42-0.49)	< 0.001
Model[a]	1 [Reference]	0.73 (0.62-0.85)	0.45 (0.42-0.49)	< 0.001
Model[b]	1 [Reference]	0.74 (0.63-0.86)	0.47 (0.44-0.51)	< 0.001
Model[c]	1 [Reference]	0.72 (0.61-0.85)	0.43 (0.39-0.46)	< 0.001
Hypertension				
Univariate model	1 [Reference]	0.81 (0.68-0.95)	0.49 (0.44-0.54)	< 0.001
Model[a]	1 [Reference]	0.76 (0.65-0.90)	0.50 (0.45-0.56)	< 0.001
Model[b]	1 [Reference]	0.78 (0.66-0.93)	0.51 (0.46-0.57)	< 0.001

Model[c]	1 [Reference]	0.68 (0.58-0.84)	0.46 (0.41-0.52)	< 0.001
Not hypertension				
Univariate model	1 [Reference]	0.56 (0.50-0.63)	0.40 (0.38-0.43)	< 0.001
Model[a]	1 [Reference]	0.56 (0.50-0.62)	0.40 (0.38-0.43)	< 0.001
Model[b]	1 [Reference]	0.54 (0.48-0.60)	0.42 (0.39-0.45)	< 0.001
Model[c]	1 [Reference]	0.51 (0.45-0.57)	0.38 (0.35-0.40)	< 0.001
Hyperlipidemia				
Univariate model	1 [Reference]	0.67 (0.54-0.84)	0.46 (0.40-0.52)	< 0.001
Model[a]	1 [Reference]	0.65 (0.52-0.81)	0.49 (0.43-0.56)	< 0.001
Model[b]	1 [Reference]	0.66 (0.53-0.82)	0.50 (0.44-0.58)	< 0.001
Model[c]	1 [Reference]	0.65 (0.51-0.83)	0.47 (0.41-0.54)	< 0.001
Not hyperlipidemia				
Univariate model	1 [Reference]	0.62 (0.56-0.69)	0.42 (0.39-0.45)	< 0.001
Model[a]	1 [Reference]	0.62 (0.56-0.68)	0.42 (0.39-0.44)	< 0.001
Model[b]	1 [Reference]	0.60 (0.54-0.66)	0.43 (0.41-0.46)	< 0.001
Model[c]	1 [Reference]	0.54 (0.48-0.61)	0.39 (0.36-0.42)	< 0.001

Demographic characteristics (age and sex) and common comorbidities (hypertension and hyperlipidemia) were stratified to analyze the association between MAFLD with overweight or obesity and combined lifestyle scores. Data are presented as odds ratios (95% CIs).

[a] Adjusted for age (continuous), sex (male or female), and education (less than high school, high school or equivalent, college or above).

[b] Further adjusted for hypertension (yes or no), CVD (yes or no), hyperlipidemia (yes or no), and diabetes (yes or no).

[c] Further adjusted for total cholesterol (continuous) and LDL-C (continuous).

Abbreviation: MAFLD, metabolic dysfunction-associated fatty liver disease; MD, metabolic dysregulation; CIs, confidence intervals.

Supplementary Table S10 Associations of each healthy lifestyle factor with risks of MAFLD and specific subtypes

Outcomes	Optimal BMI	Current nonsmoking	Current nondrinking	Adequate physical activity	Optimal sleep	Healthy diet
MAFLD	0.44 (0.42-0.47)	1.03 (0.97-1.10)	0.81 (0.75-0.88)	1.03 (0.97-1.10)	1.13 (1.06-1.21)	0.97 (0.91-1.03)
MALFD with overweight or obesity	0.11 (0.10-0.12)	1.05 (0.97-1.14)	0.78 (0.71-0.85)	1.03 (0.95-1.11)	1.21 (1.11-1.31)	0.97 (0.90-1.05)
MAFLD with diabetes	0.79 (0.67-0.93)	1.21 (0.91-1.62)	0.80 (0.64-1.01)	1.16 (0.97-1.39)	1.20 (0.88-1.64)	0.88 (0.73-1.05)
MAFLD with MD	0.41 (0.38-0.43)	1.01 (0.95-1.08)	0.79 (0.73-0.86)	1.00 (0.94-1.07)	1.13 (0.99-1.30)	0.97 (0.90-1.03)

Data are presented as odds ratios (95% CIs). The model was adjusted for age (continuous), sex (male or female) and education (less than high school, high school or equivalent, college or above), hypertension (yes or no), CVD (yes or no), hyperlipidemia (yes or no), diabetes (yes or no), total cholesterol (continuous), LDL-C (continuous), and six lifestyle factors were mutually adjusted for each other.

Abbreviation: MAFLD, metabolic dysfunction-associated fatty liver disease; MD, metabolic dysregulation; CIs, confidence intervals.

Supplementary Table S11 ORs (95% CIs) for one score increase according to three basic lifestyle factors

Lifestyle score	MAFLD	MAFLD with overweight or obesity	MAFLD with diabetes	MAFLD with MD
Score consisting of BMI, smoking, alcohol consumption	0.71 (0.69-0.74)	0.44 (0.43-0.46)	0.95 (0.86-1.04)	0.68 (0.66-0.70)
Score consisting of above three plus diet	0.78 (0.75-0.80)	0.55 (0.53-0.57)	0.95 (0.87-1.03)	0.75 (0.73-0.77)
Score consisting of above four plus physical activity	0.82 (0.80-0.85)	0.63 (0.61-0.65)	0.98 (0.91-1.06)	0.80 (0.78-0.82)
Score consisting of above five plus sleep	0.87 (0.85-0.89)	0.71 (0.69-0.73)	1.01 (0.94-1.08)	0.84 (0.82-0.86)

Data are presented as odds ratios (95% CIs). The model was adjusted for age (continuous), sex (male or female) and education (less than high school, high school or equivalent, college or above), hypertension (yes or no), CVD (yes or no), and hyperlipidemia (yes or no), total cholesterol (continuous), LDL-C (continuous).

Abbreviation: MAFLD, metabolic dysfunction-associated fatty liver disease; MD, metabolic dysregulation; CIs, confidence intervals.

Supplementary Table S12 Associations of five different baseline lifestyle scores with MAFLD and specific subtypes

Score and outcomes	Lifestyle score		
	Poor (0-2)	Intermediate (3)	Ideal (4-5)
Score consisting of BMI, smoking, alcohol consumption, diet, physical activity			
MAFLD	1 [Reference]	0.81 (0.76-0.87)	0.62 (0.58-0.67)
MAFLD with overweight or obesity	1 [Reference]	0.65 (0.60-0.70)	0.32 (0.29-0.35)
MAFLD with diabetes	1 [Reference]	1.04 (0.86-1.26)	0.90 (0.74-1.11)
MAFLD with MD	1 [Reference]	0.79 (0.73-0.84)	0.57 (0.53-0.62)
Score consisting of BMI, smoking, alcohol consumption, diet, sleep			
MAFLD	1 [Reference]	0.86 (0.80-0.93)	0.66 (0.61-0.71)
MAFLD with overweight or obesity	1 [Reference]	0.71 (0.66-0.76)	0.34 (0.32-0.37)
MAFLD with diabetes	1 [Reference]	1.07 (0.89-1.30)	0.92 (0.75-1.13)
MAFLD with MD	1 [Reference]	0.82 (0.77-0.89)	0.62 (0.57-0.66)
Score consisting of BMI, smoking, alcohol consumption, physical activity, sleep			
MAFLD	1 [Reference]	0.77 (0.71-0.83)	0.65 (0.60-0.70)
MAFLD with overweight or obesity	1 [Reference]	0.65 (0.60-0.71)	0.38 (0.35-0.41)
MAFLD with diabetes	1 [Reference]	0.89 (0.71-1.10)	0.98 (0.80-1.21)
MAFLD with MD	1 [Reference]	0.74 (0.68-0.80)	0.60 (0.56-0.65)
Score consisting of BMI, smoking, diet, physical activity, sleep			
MAFLD	1 [Reference]	0.84 (0.78-0.90)	0.70 (0.65-0.76)
MAFLD with overweight or obesity	1 [Reference]	0.65 (0.61-0.70)	0.37 (0.34-0.40)
MAFLD with diabetes	1 [Reference]	1.12 (0.93-1.35)	1.04 (0.85-1.28)
MAFLD with MD	1 [Reference]	0.83 (0.78-0.89)	0.65 (0.60-0.70)
Score consisting of BMI, alcohol consumption, diet, physical activity, sleep			
MAFLD	1 [Reference]	0.80 (0.75-0.86)	0.63 (0.58-0.67)
MAFLD with overweight or obesity	1 [Reference]	0.60 (0.56-0.64)	0.31 (0.29-0.34)
MAFLD with diabetes	1 [Reference]	0.87 (0.72-1.05)	0.84 (0.69-1.03)
MAFLD with MD	1 [Reference]	0.77 (0.72-0.83)	0.58 (0.54-0.62)

Score consisting of smoking, alcohol consumption, diet, physical activity, sleep			
MAFLD	1 [Reference]	0.92 (0.85-0.99)	0.90 (0.84-0.96)
MAFLD with overweight or obesity	1 [Reference]	0.92 (0.86-1.00)	0.88 (0.82-0.95)
MAFLD with diabetes	1 [Reference]	0.92 (0.75-1.14)	1.00 (0.82-1.22)
MAFLD with MD	1 [Reference]	0.89 (0.83-0.96)	0.86 (0.80-0.92)

Data are presented as odds ratios (95% CIs). The model was adjusted for age (continuous), sex (male or female) and education (less than high school, high school or equivalent, college or above), hypertension (yes or no), CVD (yes or no), hyperlipidemia (yes or no), diabetes (yes or no), total cholesterol (continuous), LDL-C (continuous).

Abbreviation: MAFLD, metabolic dysfunction-associated fatty liver disease; MD, metabolic dysregulation; CIs, confidence intervals.

Supplementary Table S13 Associations of healthy lifestyle score with MAFLD and specific subtypes after excluding participants with CVD at baseline

Outcomes	Lifestyle		
	Poor (0-2)	Intermediate (3-4)	Ideal (5-6)
Overall			
Univariate model	1 [Reference]	0.80 (0.74-0.87)	0.59 (0.54-0.65)
Model[a]	1 [Reference]	0.84 (0.78-0.91)	0.65 (0.59-0.71
Model[b]	1 [Reference]	0.84 (0.78-0.91)	0.66 (0.61-0.73)
Model[c]	1 [Reference]	0.81 (0.74-0.88)	0.62 (0.56-0.68)
MAFLD with overweight or obesity			
Univariate model	1 [Reference]	0.62 (0.57-0.67)	0.28 (0.26-0.31)
Model[a]	1 [Reference]	0.65 (0.60-0.70)	0.30 (0.28-0.34)
Model[b]	1 [Reference]	0.66 (0.60-0.71)	0.32 (0.28-0.35)
Model[c]	1 [Reference]	0.63 (0.58-0.69)	0.30 (0.27-0.33)
MAFLD with diabetes			
Univariate model	1 [Reference]	1.16 (0.96-1.41)	0.91 (0.72-1.13)
Model[a]	1 [Reference]	1.22 (1.00-1.49)	0.99 (0.78-1.25)
Model[b]	1 [Reference]	1.15 (0.83-1.61)	1.09 (0.74-1.60)
Model[c]	1 [Reference]	1.23 (0.86-1.76)	1.06 (0.70-1.60)
MAFLD with MD			
Univariate model	1 [Reference]	0.75 (0.70-0.81)	0.52 (0.48-0.57)
Model[a]	1 [Reference]	0.80 (0.74-0.86)	0.58 (0.53-0.63)
Model[b]	1 [Reference]	0.80 (0.74-0.87)	0.60 (0.54-0.66)
Model[c]	1 [Reference]	0.77 (0.70-0.84)	0.55 (0.50-0.61)

Data are presented as odds ratios (95% CIs).

[a] Adjusted for age (continuous), sex (male or female), and education (less than high school, high school or equivalent, college or above).

[b] Further adjusted for hypertension (yes or no), CVD (yes or no), hyperlipidemia (yes or no), and diabetes (yes or no)

[c] Further adjusted for total cholesterol (continuous) and LDL-C (continuous).

Abbreviation: MAFLD, metabolic dysfunction-associated fatty liver disease; MD, metabolic dysregulation; CIs, confidence intervals.

Supplementary Table S14 Associations of healthy lifestyle score with MAFLD and specific subtypes after excluding participants with incomplete information at baseline

Outcome	Lifestyle		
	Poor (0-2)	Intermediate (3-4)	Ideal (5-6)
Overall			
Univariate model	1 [Reference]	0.74 (0.66-0.83)	0.51 (0.44-0.58)
Model[a]	1 [Reference]	0.80 (0.71-0.90)	0.57 (0.49-0.67)
Model[b]	1 [Reference]	0.80 (0.71-0.91)	0.59 (0.51-0.68)
Model[c]	1 [Reference]	0.80 (0.71-0.91)	0.59 (0.51-0.69)
MAFLD with overweight or obesity			
Univariate model	1 [Reference]	0.62 (0.55-0.70)	0.30 (0.25-0.35)
Model[a]	1 [Reference]	0.66 (0.59-0.75)	0.34 (0.29-0.40)
Model[b]	1 [Reference]	0.68 (0.60-0.76)	0.35 (0.30-0.42)
Model[c]	1 [Reference]	0.67 (0.60-0.76)	0.35 (0.30-0.42)
MAFLD with diabetes			
Univariate model	1 [Reference]	0.89 (0.70-1.14)	0.78 (0.58-1.06)
Model[a]	1 [Reference]	0.98 (0.76-1.26)	0.95 (0.69-1.30)
Model[b]	1 [Reference]	0.90 (0.57-1.40)	0.68 (0.40-1.17)
Model[c]	1 [Reference]	0.89 (0.57-1.39)	0.69 (0.40-1.18)
MAFLD with MD			
Univariate model	1 [Reference]	0.74 (0.66-0.83)	0.51 (0.44-0.58)
Model[a]	1 [Reference]	0.80 (0.71-0.90)	0.57 (0.50-0.67)
Model[b]	1 [Reference]	0.80 (0.71-0.91)	0.59 (0.51-0.68)
Model[c]	1 [Reference]	0.80 (0.71-0.91)	0.59 (0.51-0.69)

Data are presented as odds ratios (95% CIs).

[a] Adjusted for age (continuous), sex (male or female), and education (less than high school, high school or equivalent, college or above).

[b] Further adjusted for hypertension (yes or no), CVD (yes or no), hyperlipidemia (yes or no), and diabetes (yes or no).

[c] Further adjusted for total cholesterol (continuous) and LDL-C (continuous).

Abbreviation: MAFLD, metabolic dysfunction-associated fatty liver disease; MD, metabolic dysregulation; CIs, confidence intervals.

Supplementary Table S15 Associations of healthy lifestyle score with MAFLD and specific subtypes after mental health adjustment

Outcome	Lifestyle		
	Poor (0-2)	Intermediate (3-4)	Ideal (5-6)
Overall			
Univariate model	1 [Reference]	0.79 (0.72-0.86)	0.60 (0.54-0.66)
Model[a]	1 [Reference]	0.81 (0.73-0.89)	0.63 (0.57-0.70)
Model[b]	1 [Reference]	0.81 (0.74-0.89)	0.66 (0.59-0.74)
Model[c]	1 [Reference]	0.79 (0.71-0.88)	0.63 (0.56-0.71)
MAFLD with overweight or obesity			
Univariate model	1 [Reference]	0.59 (0.54-0.65)	0.27 (0.24-0.30)
Model[a]	1 [Reference]	0.61 (0.56-0.67)	0.29 (0.26-0.33)
Model[b]	1 [Reference]	0.63 (0.57-0.69)	0.31 (0.27-0.34)
Model[c]	1 [Reference]	0.60 (0.54-0.67)	0.29 (0.25-0.33)
MAFLD with diabetes			
Univariate model	1 [Reference]	1.23 (0.99-1.52)	0.86 (0.67-1.09)
Model[a]	1 [Reference]	1.21 (0.98-1.51)	0.84(0.65-1.08)
Model[b]	1 [Reference]	0.97 (0.67-1.40)	0.95 (0.63-1.45)
Model[c]	1 [Reference]	0.97 (0.65-1.45)	0.88 (0.56-1.39)
MAFLD with MD			
Univariate model	1 [Reference]	0.73 (0.67-0.80)	0.52 (0.47-0.57)
Model[a]	1 [Reference]	0.77 (0.70-0.84)	0.57 (0.51-0.64)
Model[b]	1 [Reference]	0.78 (0.71-0.86)	0.60 (0.54-0.67)
Model[c]	1 [Reference]	0.76 (0.68-0.84)	0.57 (0.51-0.64)

Data are presented as odds ratios (95% CIs). Mental health contains 7 items, including loss of hobbies or interests, lack of energy, sudden weight loss, insomnia or poor sleep, amnesia, self-depreciation, and suicidal tendencies in the baseline survey.

[a] Adjusted for age (continuous), sex (male or female), and education (less than high school, high school or equivalent, college or above).

[b] Further adjusted for hypertension (yes or no), CVD (yes or no), hyperlipidemia (yes or no), diabetes (yes or no), mental

[c] Further adjusted for total cholesterol (continuous), LDL-C (continuous), and mental stress (yes or no).

Abbreviation: MAFLD, metabolic dysfunction-associated fatty liver disease; MD, metabolic dysregulation; CIs, confidence intervals.

Supplementary Table S16 Associations of healthy lifestyle score with MAFLD and specific subtypes after using WHO BMI cutoff

Outcome	Lifestyle		
	Poor (0-2)	Intermediate (3-4)	Ideal (5-6)
Overall			
Univariate model	1 [Reference]	0.79 (0.74-0.85)	0.64 (0.59-0.69)
Model[a]	1 [Reference]	0.84 (0.78-0.90)	0.71 (0.65-0.77)
Model[b]	1 [Reference]	0.84 (0.78-0.91)	0.74 (0.68-0.80)
Model[c]	1 [Reference]	0.82 (0.75-0.89)	0.71 (0.64-0.77)
MAFLD with overweight or obesity			
Univariate model	1 [Reference]	0.65 (0.61-0.70)	0.41 (0.37-0.44)
Model[a]	1 [Reference]	0.70 (0.64-0.75)	0.46 (0.42-0.50)
Model[b]	1 [Reference]	0.71 (0.66-0.76)	0.49 (0.44-0.53)
Model[c]	1 [Reference]	0.68 (0.63-0.74)	0.46 (0.42-0.51)
MAFLD with diabetes			
Univariate model	1 [Reference]	1.02 (0.88-1.18)	0.81 (0.68-0.95)
Model[a]	1 [Reference]	1.09 (0.94-1.27)	0.93 (0.78-1.10)
Model[b]	1 [Reference]	0.99 (0.80-1.22)	1.07 (0.84-1.37)
Model[c]	1 [Reference]	0.98 (0.78-1.22)	1.06 (0.81-1.37)
MAFLD with MD			
Univariate model	1 [Reference]	0.75 (0.70-0.81)	0.56 (0.52-0.61)
Model[a]	1 [Reference]	0.80 (0.75-0.87)	0.64 (0.59-0.70)
Model[b]	1 [Reference]	0.81 (0.75-0.87)	0.67 (0.61-0.73)
Model[c]	1 [Reference]	0.78 (0.72-0.85)	0.64 (0.58-0.70)

Data are presented as odds ratios (95% CIs). Overweight or obese was defined as BMI≥25.0 according to the classification criteria of WHO for BMI

[a] Adjusted for age (continuous), sex (male or female), and education (less than high school, high school or equivalent, college or above).

[b] Further adjusted for hypertension (yes or no), CVD (yes or no), hyperlipidemia (yes or no), and diabetes (yes or no).

[c] Further adjusted for total cholesterol (continuous), and LDL-C (continuous).

Abbreviation: MAFLD, metabolic dysfunction-associated fatty liver disease; MD, metabolic dysregulation; WHO, the World Health Organization; CIs, confidence intervals.

Supplementary Table S17 Associations of healthy lifestyle score with MAFLD and specific subtypes after using multiple imputations

Outcome	Lifestyle		
	Poor (0-2)	Intermediate (3-4)	Ideal (5-6)
Overall			
Univariate model	1 [Reference]	0.79 (0.74-0.85)	0.59 (0.55-0.64)
Model[a]	1 [Reference]	0.84 (0.78-0.90)	0.65 (0.60-0.71)
Model[b]	1 [Reference]	0.85 (0.79-0.91)	0.68 (0.62-0.74)
Model[c]	1 [Reference]	0.84 (0.78-0.91)	0.68 (0.62-0.74)
MAFLD with overweight or obesity			
Univariate model	1 [Reference]	0.62 (0.58-0.67)	0.29 (0.26-0.31)
Model[a]	1 [Reference]	0.65 (0.61-0.70)	0.32 (0.29-0.35)
Model[b]	1 [Reference]	0.66 (0.62-0.71)	0.33 (0.30-0.36)
Model[c]	1 [Reference]	0.66 (0.61-0.71)	0.33 (0.30-0.36)
MAFLD with diabetes			
Univariate model	1 [Reference]	1.09 (0.95-1.26)	0.84 (0.71-0.99)
Model[a]	1 [Reference]	1.14 (0.99-1.32)	0.92 (0.77-1.09)
Model[b]	1 [Reference]	1.09 (0.89-1.33)	1.03 (0.81-1.32)
Model[c]	1 [Reference]	1.09 (0.89-1.33)	1.03 (0.81-1.32)
MAFLD with MD			
Univariate model	1 [Reference]	0.75 (0.70-0.80)	0.52 (0.48-57)
Model[a]	1 [Reference]	0.80 (0.74-0.86)	0.59 (0.54-0.64)
Model[b]	1 [Reference]	0.81 (0.75-0.87)	0.61 (0.56-0.67)
Model[c]	1 [Reference]	0.81 (0.75-0.87)	0.61 (0.56-0.67)

Data are presented as odds ratios (95% CIs).

[a] Adjusted for age (continuous), sex (male or female), and education (less than high school, high school or equivalent, college or above).

[b] Further adjusted for hypertension (yes or no), CVD (yes or no), hyperlipidemia (yes or no), and diabetes (yes or no).

[c] Further adjusted for total cholesterol (continuous), and LDL-C (continuous).

Abbreviation: MAFLD, metabolic dysfunction-associated fatty liver disease; MD, metabolic dysregulation; CIs, confidence intervals**.**

Supplementary Figure S1 Flowchart

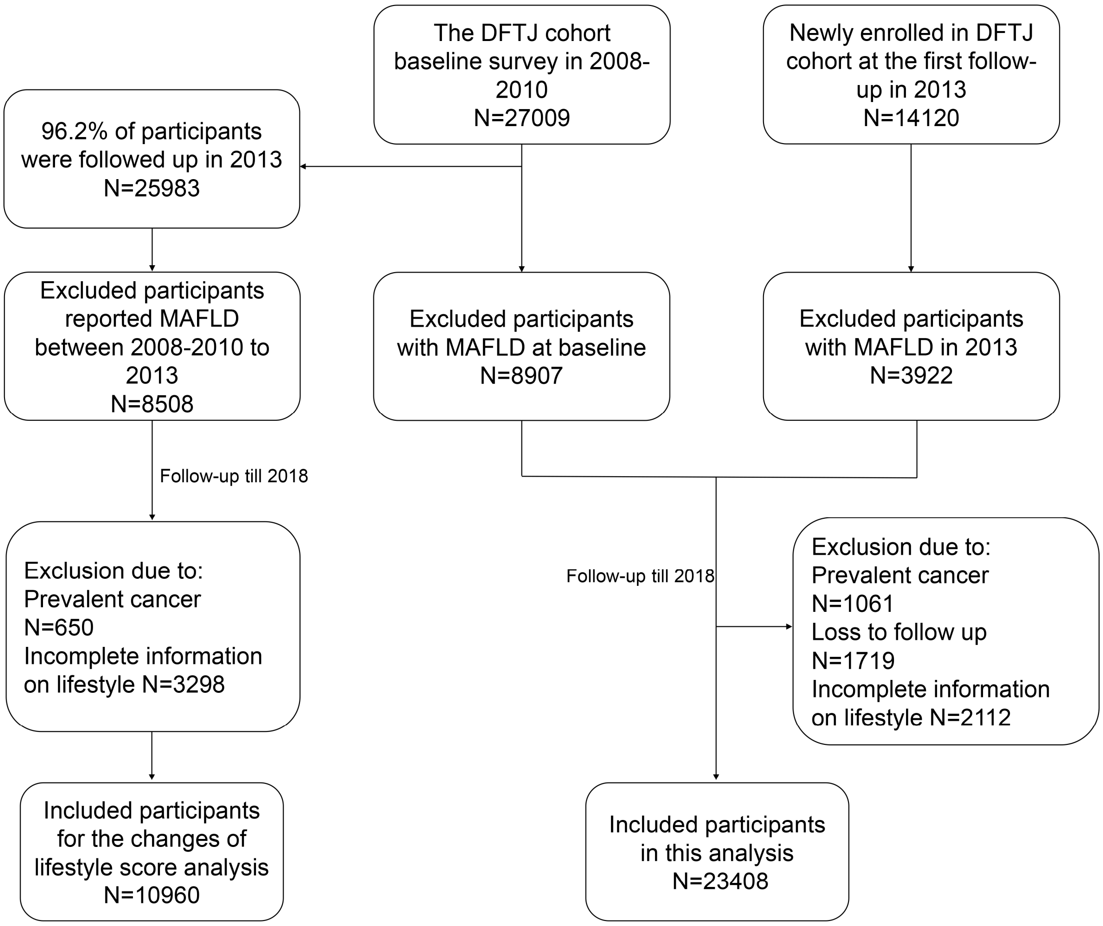

Abbreviation: MAFLD, metabolic dysfunction-associated fatty liver disease; DFTJ, Dongfeng-Tongji.

Supplementary Figure S2 Multivariate-adjusted spline curves for associations of weighted lifestyle score with the risks of MAFLD and specific subtypes

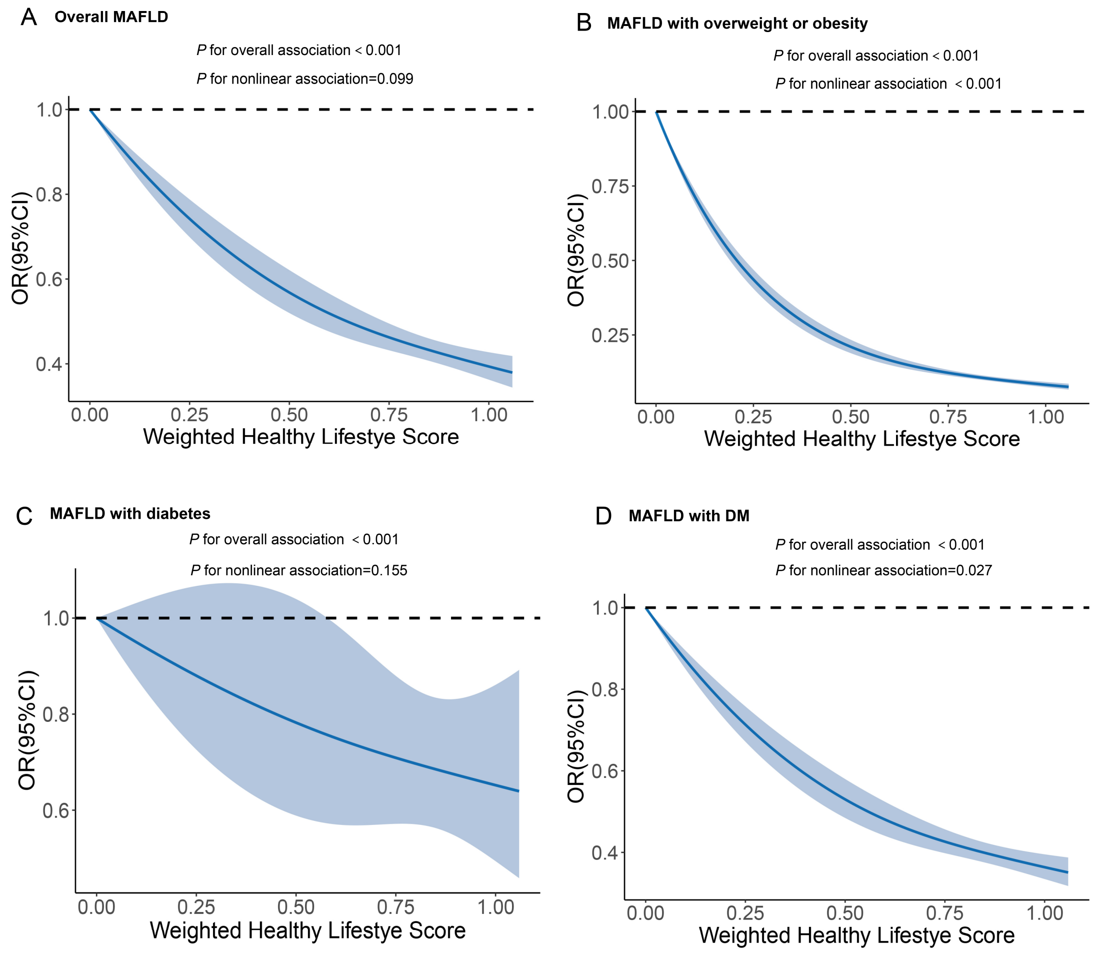

Supplementary Figure S2 Lines ORs estimated by the restricted cubic spline model, considering reference as the lowest weighted lifestyle score (0) among participants. Association of combined healthy lifestyle score with (A) overall MAFLD, (B) MAFLD with overweight or obesity, (C) MAFLD with diabetes, and (D) MAFLD with MD. ORs were represented by the solid line and 95% CIs were represented by the shading area. Adjusted covariates included age (continuous), sex (male vs female) and education (less than high school, high school or equivalent, college or above), hypertension (yes or no), CVD (yes or no), hyperlipidemia (yes or no), diabetes (yes or no), total cholesterol (continuous), and LDL-C (continuous).

Abbreviation: MAFLD, metabolic dysfunction-associated fatty liver disease; MD, metabolic dysregulation; ORs, odds ratios; CIs, confidence intervals.

Supplementary Figure S3 Stratified Analysis of association of weighted healthy lifestyle score with an incident of MAFLD and its specific subtypes

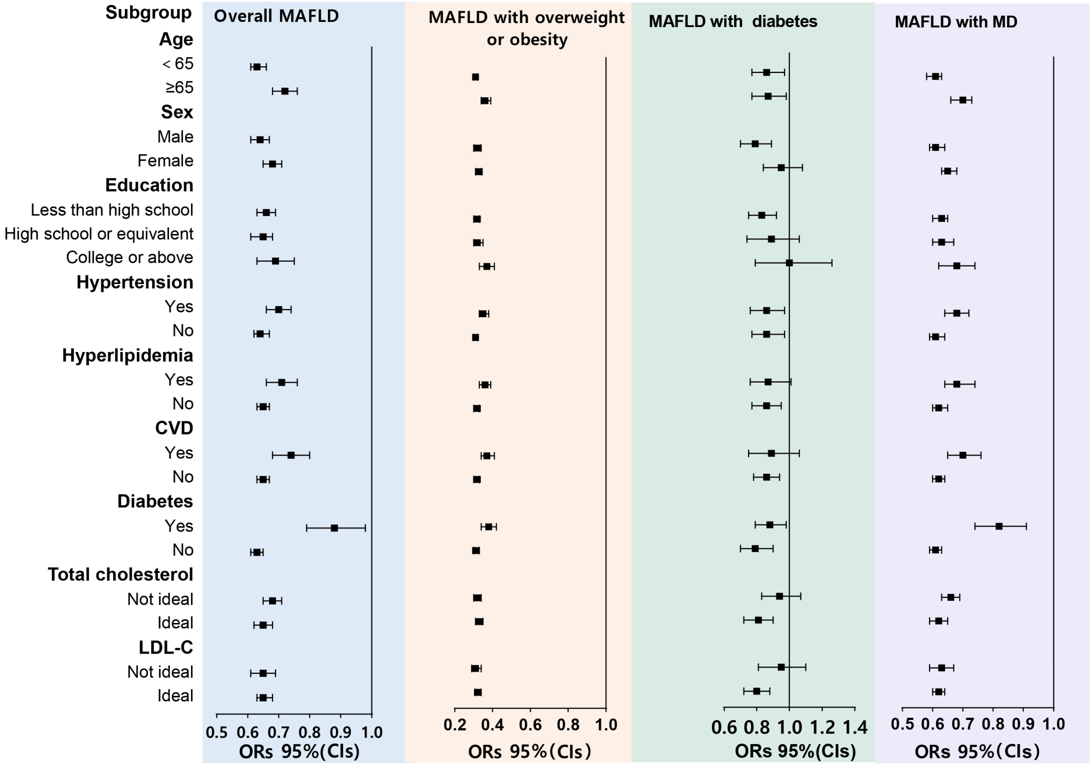

Supplementary Figure S3 Association of weighted healthy lifestyle score with an incident of MAFLD and its specific subtypes in participants stratified by demographic, metabolic, and MAFLD-related features. Adjusted for age (continuous), sex (male or female) and education (less than high school, high school or equivalent, college or above), hypertension (yes or no), CVD (yes or no), hyperlipidemia (yes or no), diabetes (yes or no), total cholesterol (continuous), and LDL-C (continuous). Each group adjusted for the other covariates except itself.

Abbreviation: MAFLD, metabolic dysfunction-associated fatty liver disease; MD, metabolic dysregulation; ORs, odds ratios; CIs, confidence intervals.

Reference

1. Wang F, Zhu J, Yao P, et al: Cohort Profile: the Dongfeng-Tongji cohort study of retired workers**.** *Int J Epidemiol* **2013**, *42***:**731-740.
2. Janjua M, Knuiman M, Divitini M, et al: Alcohol Consumption and Cardiovascular Outcomes in Patients With Nonalcoholic Fatty Liver Disease: A Population-Based Cohort Study**.** *Hepatol Commun* **2022**, *6***:**526-534.
3. Collaborators GBDA: Alcohol use and burden for 195 countries and territories, 1990-2016: a systematic analysis for the Global Burden of Disease Study 2016**.** *Lancet* **2018**, *392***:**1015-1035.
4. Peng H, Pan L, Ran S, et al: Prediction of MAFLD and NAFLD using different screening indexes: A cross-sectional study in U.S. adults**.** *Front Endocrinol (Lausanne)* **2023**, *14***:**1083032.

5. Lloyd-Jones DM, Allen NB, Anderson CAM, et al: Life's Essential 8: Updating and Enhancing the American Heart Association's Construct of Cardiovascular Health: A Presidential Advisory From the American Heart Association**.** *Circulation* **2022**, *146***:**e18-e43.
6. Wang Q, Guo Q, Zhou L, et al: Associations of Baseline and Changes in Leukocyte Counts with Incident Cardiovascular Events: The Dongfeng-Tongji Cohort Study**.** *J Atheroscler Thromb* **2022**, *29***:**1040-1058.
7. Han X, Wei Y, Hu H, et al: Genetic Risk, a Healthy Lifestyle, and Type 2 Diabetes: the Dongfeng-Tongji Cohort Study**.** *J Clin Endocrinol Metab* **2020**, *105*.
8. Lu Q, Zhang Y, Geng T, et al: Association of Lifestyle Factors and Antihypertensive Medication Use With Risk of All-Cause and Cause-Specific Mortality Among Adults With Hypertension in China**.** *JAMA Netw Open* **2022**, *5***:**e2146118.
